# Photosystem-II D1 protein mutants of *Chlamydomonas reinhardtii* in relation to metabolic rewiring and remodelling of H-bond network at Q_B_ site

**DOI:** 10.1038/s41598-018-33146-y

**Published:** 2018-10-03

**Authors:** Amina Antonacci, Maya D. Lambreva, Andrea Margonelli, Anatoly P. Sobolev, Sandro Pastorelli, Ivo Bertalan, Udo Johanningmeier, Vladimir Sobolev, Ilan Samish, Marvin Edelman, Vesa Havurinne, Esa Tyystjärvi, Maria T. Giardi, Autar K. Mattoo, Giuseppina Rea

**Affiliations:** 1 Institute of Crystallography, National Research Council of Italy, Via Salaria Km 29,3 00015, Monterotondo Stazione, Rome Italy; 2 Institute of Chemical Methodologies, National Research Council of Italy, Via Salaria km 29,3 00015, Monterotondo Stazione, Rome Italy; 3 Present Address: Neotron S.p.a., Santa Maria di Mugnano, Modena, Italy; 4 Martin-Luther-University, Plant Physiology Institute, Weinbergweg 10, D-06120 Halle Saale, Germany; 5 Department of Plant and Environmental Sciences, Weizmann Institute of Science, Rehovot, Israel; 6 Present Address: Amai Proteins Ltd., 2 Bergman St., Rehovot, Israel; 7 Department of Biochemistry/Molecular Plant Biology, FI-20014, University of Turku, Turku, Finland; 8 The Henry A Wallace Beltsville Agricultural Research Centre, United States Department of Agriculture, Sustainable Agricultural Systems Laboratory, Beltsville, Maryland 20705 USA

## Abstract

Photosystem II (PSII) reaction centre D1 protein of oxygenic phototrophs is pivotal for sustaining photosynthesis. Also, it is targeted by herbicides and herbicide-resistant weeds harbour single amino acid substitutions in D1. Conservation of D1 primary structure is seminal in the photosynthetic performance in many diverse species. In this study, we analysed built-in and environmentally-induced (high temperature and high photon fluency – HT/HL) phenotypes of two D1 mutants of C*hlamydomonas reinhardtii* with Ala250Arg (A250R) and Ser264Lys (S264K) substitutions. Both mutations differentially affected efficiency of electron transport and oxygen production. In addition, targeted metabolomics revealed that the mutants undergo specific differences in primary and secondary metabolism, namely, amino acids, organic acids, pigments, NAD, xanthophylls and carotenes. Levels of lutein, β-carotene and zeaxanthin were in sync with their corresponding gene transcripts in response to HT/HL stress treatment in the parental (IL) and A250R strains. D1 structure analysis indicated that, among other effects, remodelling of H-bond network at the Q_B_ site might underpin the observed phenotypes. Thus, the D1 protein, in addition to being pivotal for efficient photosynthesis, may have a moonlighting role in rewiring of specific metabolic pathways, possibly involving retrograde signalling.

## Introduction

Oxygenic photosynthesis evolved at least 2.4 billion years ago, making oxygenic life possible on earth and becoming an essential energy resource. Multisubunit chlorophyll–protein complexes heralded by two photosystem (PS) reaction centres, PSII and PSI, catalyse electron transfer from water to NADP^+^ using energy harvested from sunlight. At the core of the PSII reaction centre is a heterodimer made of two proteins, D1 and D2, with their redox cofactors^1,2^. The D1 protein is characterized by a rapid, photon-flux-dependent turn-over^3,4^ and directly mediates photosynthetic electron transport and oxygen evolution^1,5^.

Structural resolution of the PSII reaction centre (RC) provided atomic details and structural/functional relationships of the D1 and D2 protein heterodimer including its bound redox cofactors involved in light-driven photochemical reactions^6–11^. It also enabled analysis of the effects of amino acid substitutions in D1 on photosynthesis and response of such mutants to environmental extremes in *Chlamydomonas*^12–15^. Earlier, it had been established that herbicide (e.g., atrazine, DCMU)-resistant weeds had single amino acid residue substitutions in their D1 protein^16–18^ while D1 protein synthesis was unaffected^19^. Besides modification of herbicide sensitivity, site-specific mutations in the DE-stromal-loop of D1 often resulted in severe reduction of mutant photoautotrophic growth and photosynthetic performance, due mainly to impairment of the electron transfer between primary (Q_A_) and secondary (Q_B_) PSII quinones^20,21^. Mutations involving I248T, S264G, S264A, A251V and F211S substitutions in plants, cyanobacteria and green algae made them susceptible to photoinhibitory light conditions^20,22–25^. Notably, soybean cell line STR7 with S268P D1 mutation was found unusually tolerant to high temperature (HT)^26,27^ and high light (HL)^2^, and this phenotype was found associated with higher unsaturation of fatty acids. Similar alterations in lipid composition and chloroplast ultrastructure were evident in several triazine-resistant weeds and in aquatic *Spirodela* cultivated on a sublethal concentration of the herbicide atrazine^28^.

Changes in flexible domains involving D1-209 and D1-212 residues enabled adaptation of *Synechocystis* sp. PCC6803 to the ambient temperature^29^ while the adjacent D1-208 residue was found to control electron transfer gating in photosynthetic reaction centers^30^. Moreover, the same cyanobacterium strain responded to low-oxygen exposure by up-regulating, among others, the *psbA1* gene that encodes D1, which was considered as a part of stress-adaptation process^31^. Interestingly, the DE-loop of D1 has been suggested as a heat-sensitive cleavage site that is protected in the presence of photosynthetic herbicides^32^. Thus, studies with a number of biological systems harbouring specific D1 amino acid mutations have provided knowledge on D1 structural motifs that regulate PSII function, enable plant acclimation to extreme temperatures (as in thermophiles) and resilience to herbicide-resistant biotypes.

Metabolic consequences of single point mutations at the Q_B_ binding site of D1, the Q_B_ site being the major herbicide binding niche, are therefore of major interest in terms of both structural and regulatory functions. Electron transfer from Q_A_ to Q_B_ is the rate determining step on the reducing side of PSII and, as such, the Q_B_ binding site on D1 is an advantageous location to examine downstream genetic control of physiological stress-coping phenomena. Site-directed mutants S264K and A250R were selected and compared to the IL parent strain. These two *C*. *reinhardtii* D1 mutants host a modified DE-loop region in which a native amino acid is replaced with one having a bulkier side chain. Also, the A250R and S264K mutants differ in their sensitivity to triazine herbicides, A250R is highly sensitive while S264K is resistant^12,25,33^.

Based on the above characteristics of the PSII D1 protein, we hypothesized that further studies of some of its mutants should provide insights into its moonlighting functions ‘in planta’ using *Chlamydomonas*. Therefore, we analysed and evaluated S264K and A250R mutants together with the IL parent strain for photosynthetic performance, their primary and secondary metabolite profiles, and gene expression patterns related to carotenoids and PQ/tocopherol in the absence and presence of a combination of HL/HT conditions that mimic a natural stress environment. We demonstrate here that in addition to photochemistry, the photosynthetic reaction centre D1 protein is likely associated with reprogramming pigment and metabolic pathways in *C*. *reinhardtii*.

## Results

### Features of *C*. *reinhardtii* D1 mutants under physiological conditions

#### Growth rate and photosynthetic performance

**Figure 1:**
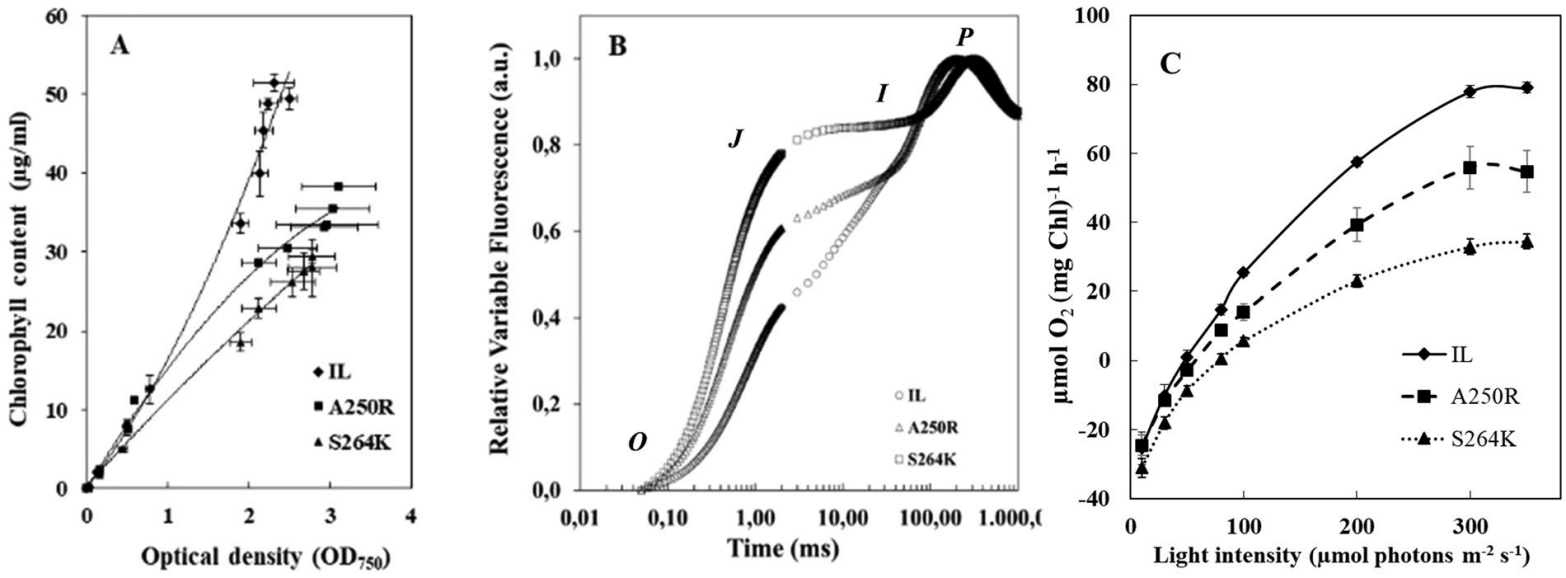
Mixotrophic growth, fluorescence measurements and light dependency curves of the photosynthetic activity in the *C*. *reinhardtii* IL, A250R and S264K strains. (**A**) Time course of Chl accumulation for 168 h of growth, expressed as a ratio of Chl content^57^ and optical density at 750 nm. The difference between the mutants and the parent strain gradually increased with time; average of three independent experiments, ± SE, n = 3. (**B**) Curves of relative variable fluorescence, (*V*(*t*) = (*F*_*t*_ − *F*_0_)*/*(*F*_*m*_ − *F*_0_)). Average curves of four technical repetitions are shown; the O-J-I-P characteristic points are indicated. (**C**) The rate of oxygen evolution was measured as a function of the light intensity (from 0 to 350 µmol m^−2^ s^−1^, provided by red LED Light Source) at 25 °C and in the presence of 10 mM NaHCO_3_. The rate of oxygen production at each light intensity is an average of 2 min continuous recording; at least three biological repetitions were done per each strain and each curve represents an average of three experiments ± SE, n = 6.

The growth rate of parent strain *IntronLess* (IL) and D1 mutants A250R and S264K followed a similar trend, but the two mutants accumulated less chlorophyll (Chl) in comparison to IL (Figs 1A and S1). The differences in total Chl content became evident during the early exponential growth phase (OD_750_ ~0.4), when A250R and S264K, respectively, accumulated 60% and 42% of the Chl*a* + *b* per cell of the reference strain IL (Table S1), and maximized at the late growth phase (Figs 1A and S1B).

Chl *a* fluorescence induction transient (*OJIP*) was used to evaluate the electron transfer efficiency of the *Chlamydomonas* strains, and relative variable fluorescence curves, *V*_*t*_, were calculated^34^ to directly compare the Q_A_^−^ accumulation dynamics in the three strains. Amino acid substitutions A250R and S264K led to an impairment of Q_A_^−^ reoxidation, evident by the increase in the *V*_*J*_ fluorescence levels compared to the parent line IL (Fig. 1B). Likewise, the efficiency of electron transfer rate between Q_A_ and Q_B_ quinone acceptors in PSII, *1-V*_*J*_, followed the sequence IL > A250R > S264K (Table S1). The substitution Ser → Lys at position 264 led to a 50% reduction of the mutant *1-V*_*J*_ compared to IL (0.56 ± 0.03 for IL *versus* 0.23 ± 0.01 for the mutant) as well as to a delay in reaching the maximum fluorescence level (time to reach *P* step in Fig. 1B). On the other hand, the maximum quantum yield of PSII photochemical reaction was less affected by the mutations. In fact, the reduction in the *F*_*v*_*/F*_*m*_ ratio was about 10% in S264K mutant and about 5% in A250R compared to IL (Table S1).

Next, the rate of O_2_ evolution was measured as a function of light intensity to evaluate the PSII functionality (Fig. 1C). The photosynthetic reactions in the three strains saturated at a similar light intensity (around 300 µmol m^−2^ s^−1^). The maximum rate of O_2_ evolution in A250R and S264K was about 70% and 40% compared to IL, respectively. The mutants were similarly reduced in the photosynthetic efficiency as seen by regression of the linear part of the light-saturated curve of O_2_ evolution. Notably, both the A250R and S264K mutants were impaired in the PSII electron transfer efficiency and pigment accumulation as compared to IL, with S264K relatively more impaired than A250R (Table S1). These data are in tune with the lower photosynthetic efficiency and higher light compensation point in the A250R and S264K mutants compared to IL (Table S1). The dark respiration rate did not differ very much among the strains (Table S1), suggesting that the increase in the light compensation point observed in the mutants is due mainly to alteration in the performance of linear electron transport and light harvesting.

### Targeted metabolomics

#### Profiling of carotenoids

Pigment profiles of photosynthesizing organisms are related to photosynthetic performance and capacity. Differential content of chlorophylls prompted us to analyse the profiles of other pigments. Both mutants were distinguishable from the parent strain in regard to the accessory pigments’ HPLC profiles, mainly xanthophylls, lutein and β-carotene. Specifically, the content of violaxanthin, antheraxanthin, zeaxanthin, β-carotene and lutein was significantly impacted in both the mutants *versus* the IL strain (Fig. S2).

#### NMR-based metabolite patterns

NMR spectroscopy of extracts from the control and mutant lines was performed to seek any further metabolic shifts. Out of 139 NMR signals, the forty most intense were assigned to 21 known metabolites (Table S2). Identified metabolites included nine free amino acids (Ala, Glu, Ile, Leu, Lys, Phe, Thr, Tyr, and Val), six organic acids (acetic, formic, fumaric, lactic, malic, and succinic), three nucleosides and their derivatives (NAD, AMP, uracil) and three others (maltodextrin, putrescine, DHU). Phe and Tyr levels in the control were half of that in the two D1 mutants and a similar trend was observed for Ala and Lys too, while the level of Thr was higher in the parent IL. Glu, Ile, Leu and Val levels were found to be similar in all the three lines. In A250R, the levels of putrescine, malic acid and acetic acid were higher than in the control line but no substantial differences were observed in the levels of DHU, uracil, AMP, and the organic acids: formic, fumaric, lactic, and succinic. Putrescine and malic acids were also at higher levels in the S264K mutant than in the control line, as were AMP, DHU, formic acid, fumaric acid, and succinic acid. Maltodextrin levels were similar in the control and S264K, whereas its level was drastically reduced in A250R. Finally, the levels of NAD were considerably lower in the two mutants compared to the control (Table S2).

#### Correlation analysis between NMR metabolites and pigment levels

**Figure 2:**
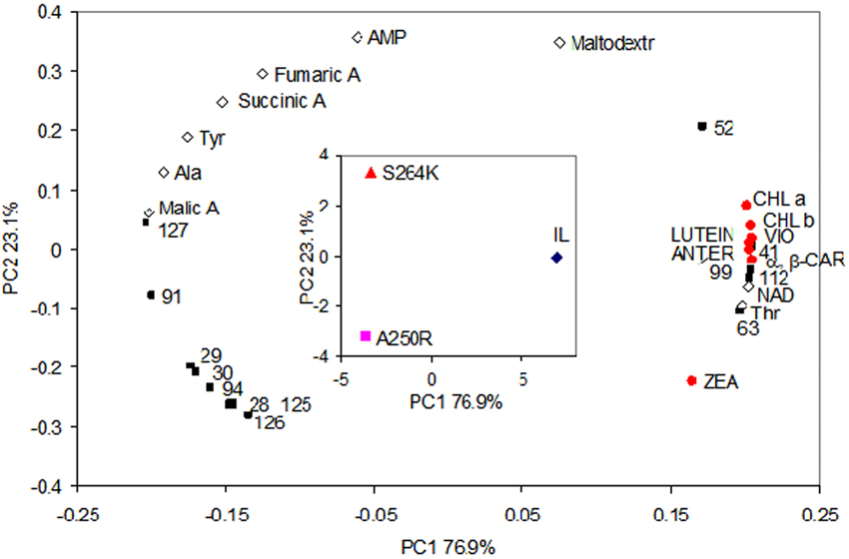
PCA of NMR data and pigments’ content. Red filled circles, pigments; black filled quadrates, unassigned NMR signals; black empty rhombuses, assigned NMR signals. Insert: scores plot.

PCA was applied to the mean values of 22 NMR signals selected by ANOVA together with 8 pigments to reveal effective correlations (Fig. 2). The principal components, which are linear combinations of original variables, correspond to the mutually orthogonal directions with a maximum variance of data. A few first principal components describe the entire system and simplify the data exploration. The score plot in the centre of the loadings plot is highlighted (Fig. 2). The parent IL line and the two mutants separated along the PC1 axis. The variables involved in this separation are those with the highest or lowest PC1 loadings that correspond to the contribution of each variable to PC1. Positive loadings correspond to a positive correlation between original variables and principal components, therefore a higher PC1 score of IL with respect to mutants is in agreement with higher values of original variables with positive loadings. The PC1 loadings of almost all pigments (red filled circles) are the highest among all variables indicating that the level of pigments in IL is significantly higher than in the two mutants. All NMR signals with loadings similar to those of pigments are potentially correlated with each other and have the highest level in the IL line, thus NAD, Thr and unassigned NMR signals 41, 63, 99 and 112 are correlated with pigments. Among the unassigned signals, 41 (6.095 ppm) most probably belong to the ribose ring in nucleosides, whereas the identity of 63, 99, and 112 remains unknown.

The variables with the lowest PC1 loadings (malic acid, Tyr, Ala, and unassigned 91) were also separated between IL and the D1 mutants, being at lowest levels in the IL line. Finally, the separation between A250R versus S264K along the PC2 axis was due to AMP and maltodextrin which showed the highest PC2 loadings. The unassigned NMR signals 28, 94, 125 and 126, have the lowest PC2 loadings and, therefore, contributed to separation between A250R and S264K, although the levels of the 126 and 28 signals were not significantly different based on ANOVA. The correlation between NAD and pigment levels is interesting and likely related to photosynthetic performance and capacity, since NAD is a precursor of NADP coenzyme involved in photosynthesis.

### Response of *C*. *reinhardtii* strains to HL/HT

#### Photosynthetic performance

**Figure 3:**
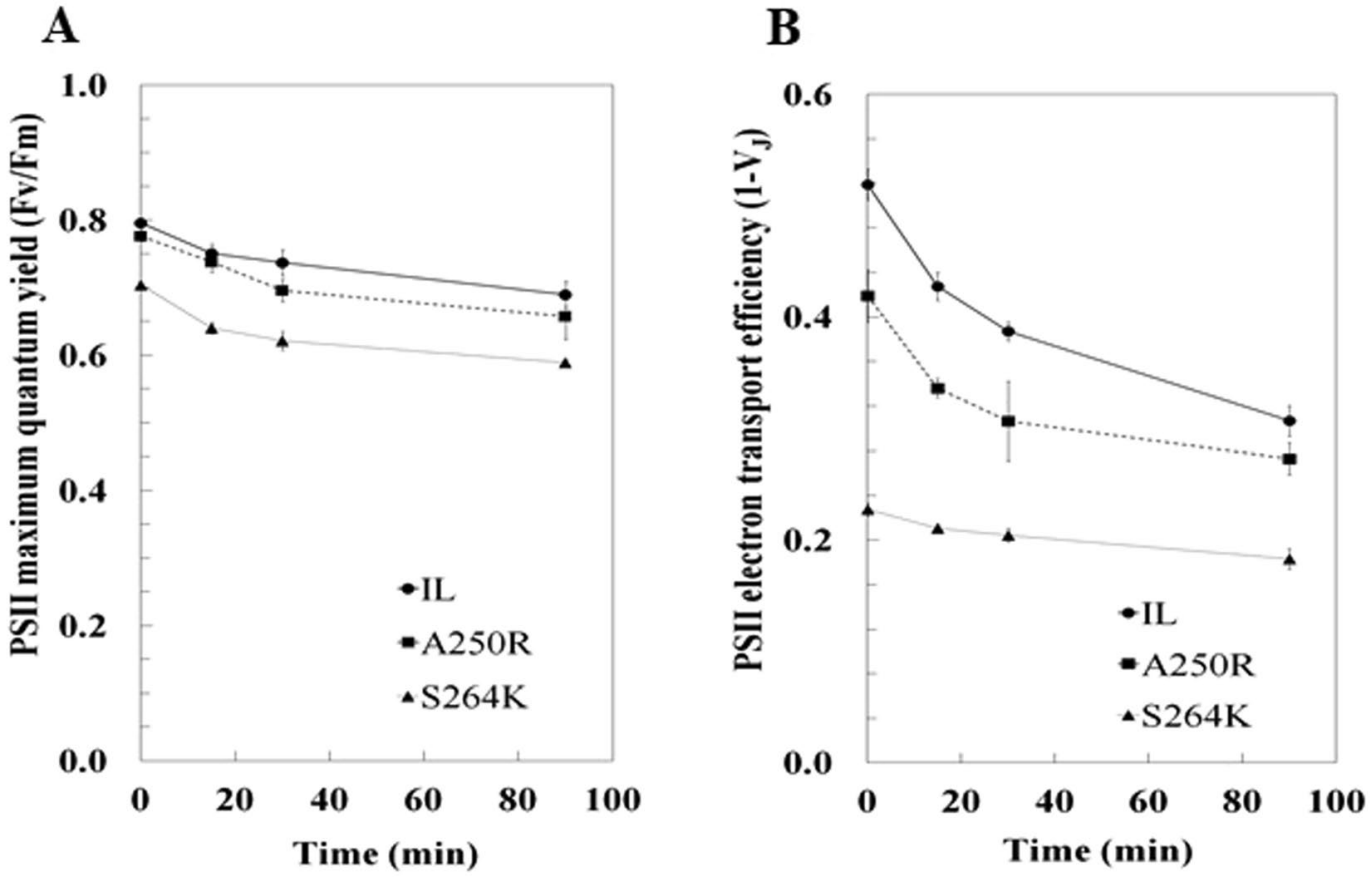
Reduction of PSII performance of *C*. *reinhardtii* strains during the HL/HT treatment. (**A**) The maximum quantum yield of PSII photochemical reaction (F_v_/F_m_ = (F_m_ − F_0_)/F_m_) and (**B**) electron transport efficiency [1 − V_J_ = (1−(F_J_ − F_0_)/(F_m_ − F_0_)] were calculated for cultures exposed for 0, 15, 30 and 90 min to 1000 µmol m^−2^ s^−1^ light intensity at 37 °C. At the corresponding times, samples were harvested also from cultures kept under normal growth conditions as a control. The values of F_v_/F_m_ and 1-V_J_ for each strain at time 0 on panel C and D, respectively, represent average from all control values during the 90 min experiment, ± SE, n = 24. All other reported values are an average of three independent experiments ± SE, n = 6. P ≤ 0.05 (Mann-Whitney U Test).

We determined the response of parent IL strain and the two D1 mutants to simultaneous exposure to double stress: high photon fluency rate (1000 µmol m^−2^ s^−1^) and high temperature (37 °C) (HL/HT) for 15, 30 and 90 min. Control samples were harvested at the same intervals from duplicate cultures held in un-stressed conditions. In response to HL/HT treatment, the mutants were slightly affected in the maximum quantum yield of PSII photochemical reaction, the reductions ranged from 13% to 16% between the mutants and the control strain (Fig. 3A). However, the difference in the PSII electron transport efficiency between the two mutants was pronounced, differentiating the two mutants under unstressed conditions (Table S1). Upon HL/HT treatment, alteration of *1-V*_*J*_ in A250R was similar to that found in the parent IL strain, displaying ~40% inhibition of PSII electron transport efficiency at the end of the treatment (*1-V*_*J*,_ Fig. 3B). In contrast, the S264K mutant, which is physiologically more compromised in the electron transfer capacity, displayed a smaller (20%) reduction in the *1-V*_*J*_ parameter compared to IL under HL/HT conditions.

#### Targeted metabolomics

**Figure 4:**
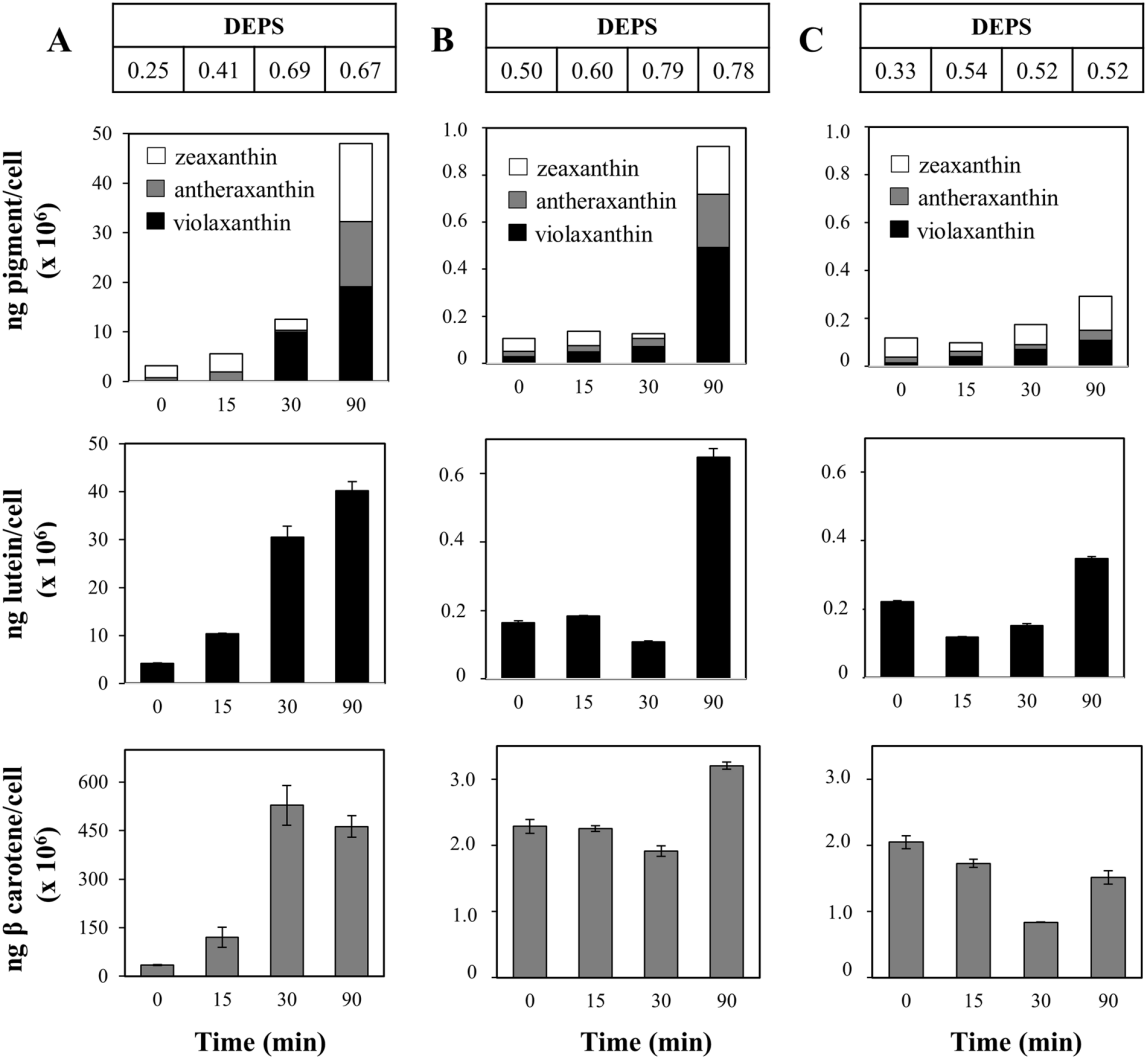
Quantitative analyses of photosynthetic pigments by HPLC. Pigment content per cell and DEPS (Z + A)/(V + A + Z) in *C*. *reinhardtii* IL, A250R and S264K strains under HL/HT condition. Contents of violaxanthin, antheraxanthin and zeaxanthin (upper), lutein (middle) and β carotene (lower) for IL (panel A), A250R (panel B) and S264K (panel C) strains. Each histogram is an average of three biological repetitions ± SE, n = 9.

Exposure to HL/HT stress resulted in a gradual rise in the total content of pigments involved in the xanthophyll cycle (violaxanthin, V; antheraxanthin, A; zeaxanthin, Z), with a ~15-fold and ~4-fold increase in IL and A250R, respectively, and a ~3-fold increase in S264K after 90 min of exposure (Fig. 4). The xanthophyll cycle, estimated by calculation of the de-epoxidation state (DEPS), was active in all the three phenotypes but the accumulation of the photoprotective pigment zeaxanthin was differential and occurred with different kinetics. In addition, the HL/HT treatment led to parallel increments in the relative amounts of β-carotene and lutein in IL and A250R (Fig. 4A,B). However, the response of the S264K mutant to imposed stress was weaker in eliciting xanthophylls and lutein, but β-carotene levels were decreased (Fig. 4C).

**Figure 5:**
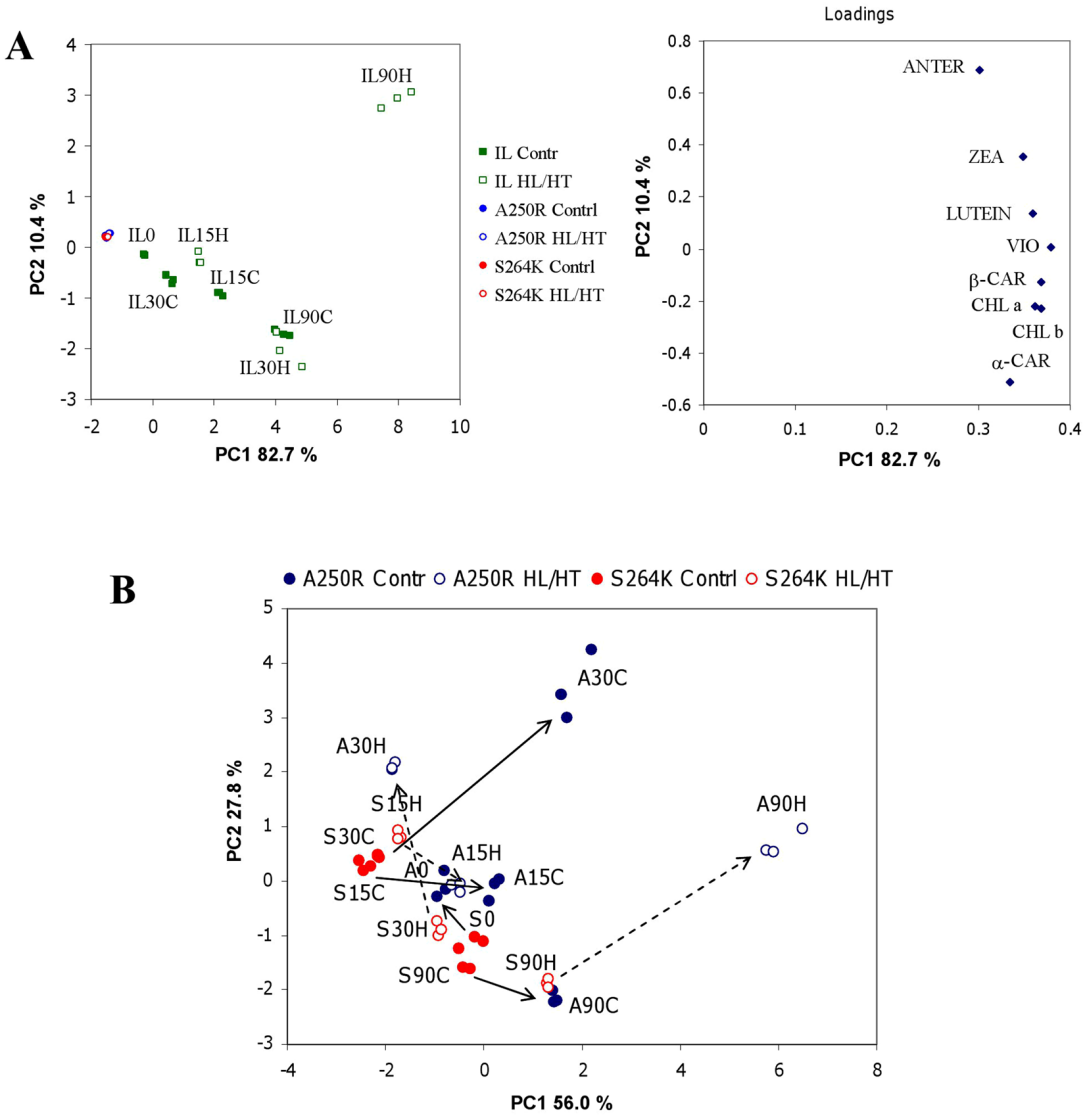
(**A**) PCA scores (left) and loadings plots (right). (**B**) PCA scores plot for A250R and S264K samples. Solid and dotted arrows indicate trends for the control and HL/HT, respectively. A15H, A30H, A90H: data of A250R after 15, 30 and 90 min of HL/HT treatment. S15H, S30H, S90H: data of S264K after 15, 30 and 90 min of HL/HT treatment. A0, A15C, A30C, A90C: control A250R samples after 0, 15, 30 and 90 min. S0, S15C, S30C, S90C: control S264K samples after 0, 15, 30 and 90 min.

Different temporal trends were apparent between control and HL/HT-treated IL line with an abundant increase in antheraxanthin and zeaxanthin levels (Fig. 5A, 90 min). The variation of pigment levels in the A250R and S264K mutants was relatively small versus IL. Almost all samples from the mutants were located in a very small region of the PCA score plot. An expanded plot region where A250R and S264K sample scores are located is shown in Fig. S3. The expanded region showed a drastic increase in antheraxanthin and zeaxanthin levels in HL/HT-treated A250R line at 90 min as compared to the IL line. However, this was not observed for similarly treated S264K line.

All pigment levels seemed correlated to some extent, with the most significant correlation observed for β-carotene/α-carotene and Chl*a* and Chl*b* (Fig. S4). Interestingly, the ratio of β-carotene/α-carotene remained constant in all the samples except for IL-90 min HL/HT, and S264K-90 min HL/HT (Fig. S4). The deviation of β-carotene/α-carotene ratio in these cases is indicative of variation in metabolism and deserves further investigation.

#### PCA highlights differences between the A250R and S264K mutants

The responses under control conditions *versus* HL/HT-treatment were different in A250R: small differences were apparent at 15 min and became substantial after 30 and 90 min of exposure. Almost all pigments in HL/HT samples decreased at 30 min in comparison to control but were significantly elevated in 90-min samples with the exception of chlorophylls (Fig. 5B). The A250R/S264K ratios for each pigment are given in Table S3. The arrows pointing to the right in Fig. 5B (for example, controls or HL/HT at 90 min) indicate that the pigment levels in the A250R mutant are higher than in the S264K mutant. In fact, the corresponding ratios (Table S3) are > 1 for almost all the analysed pigments except Chl*b* following HL/HT treatment. The two arrows (S0 → A0 and S30H → A30H) pointing to the left side of the plot in Fig. 5B indicate a tendency for higher pigment levels in S264K samples in comparison to A250R *versus* the corresponding control (0 min) and HL/HT (30 min) samples. Ratios shown in Table S3 confirm this analysis with a few exceptions: zeaxanthin and β-carotene in control T0, and antheraxanthin and carotenes in HL/HT 30 min samples are more abundant in A250R than in the S264K line.

### Comparative gene expression profiles dictated by HL/HT treatment

#### Carotenoid pathway gene expression profile

**Figure 6:**
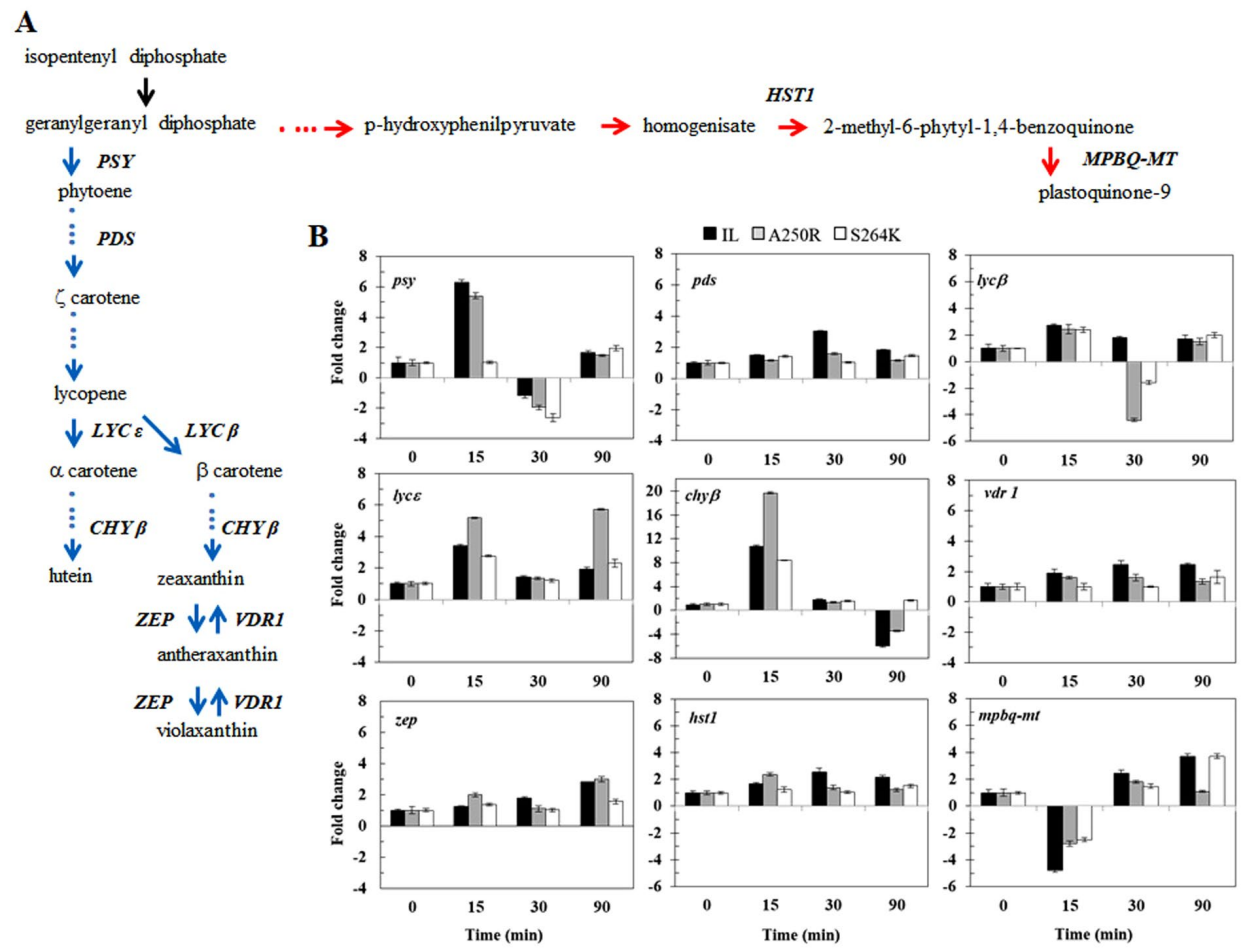
Gene expression analysis of *C*. *reinhardtii* IL, A250R and S264K strains. (**A**) Schematic representation of carotenoids (blue arrows) and PQ (red arrow) biosynthetic pathways (adapted from^38,39^). In *italics* are the enzymes analysed by qRT–PCR of samples under HL/HT treatment. (**B**) Gene expression analyses of carotenoids and PQ/tochopherol biosynthetic pathway genes in response to HL/HT treatment. Time course of mRNA expression levels of phytoene synthase (*psy*), phytoene desaturase (*pds*), lycopene β-cyclase (*lycβ*), lycopene ε-cyclase (*lycε*), carotene β-hydroxylase (*chyβ*), violaxanthin de-epoxidase (*vdr*), zeaxanthin epoxidase (*zep*), homogentisate solanesyl transferase (*hst1*) and methylphytyl benzoquinone methyltransferase (*mpqb-mt*) by qRT–PCR. *rack1* gene was used as the endogenous control. The results are an average of three biological replicates, ± SE, n = 9.

Next, we quantified changes in the mRNA levels of genes involved in the biosynthesis of carotenoid/PQ/tocopherol pigments in response to HL/HT treatment using Real-Time PCR (Fig. 6A, blue arrows). The *psy* gene, encoding phytoene synthase, which catalyses conversion of geranylgeranyl diphosphate to phytoene, was induced within 15 min in IL and A250R lines (by 6.3 and 5.4-fold, respectively) but not in the S264K line. Thereafter, *psy* was drastically down-regulated at 30 min in all the three lines and recovered after 90 min to low but significantly higher levels than the zero-point controls following HL/HT treatment (Fig. 6). In comparison, the *pds* gene, encoding phytoene desaturase and catalysing the synthesis of ζ-carotene, was less reactive to HL/HT treatment. Only the IL line showed a 3-fold induction of *pds* mRNA after 30 min exposure to HL/HT treatment (Fig. 6).

The genes involved in lycopene cyclization and biosynthesis of lutein and zeaxanthin, namely, *lyc*-β, *lyc*-ε and *chy*-β, registered unique expression patterns. All the three genes were strongly up-regulated in all the three lines but to different degrees: At 15-min HL/HT treated IL samples registered increase in *lyc*-β, *lyc*-ε and *chy*-β by 2.7, 3. 4 and 10.8-fold, respectively; in A250R, the increase was 2.4, 5.2 and 19.7-fold, respectively; and in S264K, the increase was 2.4, 2.8 and 8.4-fold, respectively (Fig. 6). Subsequently, *lyc-β* mRNA levels decreased in all the lines, but more so in the two mutants: at 30 min of HT/HL, the levels decreased in A250R by 4.4-fold and in S264K by 1.6-fold (Fig. 6B). By 90 min of HL/HT treatment, *lyc*-β mRNA levels recovered to being slightly but significantly higher than in the untreated control lines. Expression of *lyc*-ε, whose protein product catalyses the synthesis of α carotene, decreased to similar levels at 30 min exposure and then again differentially increased at 90 min. These changes occurred in both mutants as compared to the IL line but clearly, A250R had the highest expression levels at 15 and 90 min exposure to HL/HT (Fig. 6B). Similarly, the expression pattern of *chy*-β mRNA, whose protein product catalyses a necessary step in the conversion of carotene to the carotenoid lutein, was unique. The extent of increase in its relative mRNA levels at 15-min exposure to HL/HT was the highest of all the tested genes in the three lines; the maximum registered was in the A250R mutant. The relative gene expression in all the lines at 30-min HT/HL treatment reverted to levels closer to that at 0 time, decreasing further to negative values in 90-min treated samples in the IL and A250R lines but not in the S264K line (Fig. 6).

Xanthophyll inter-conversion pathway, zeaxanthin ↔ antheraxanthin ↔ violaxanthin genes, *vdr1* and *zep*, were relatively steadier with slow but steady increases in expression in the IL control and A250R mutant in response to HL/HT treatment (Fig. 6). Both these genes in S264K mutant showed only a slight increase in response to the HT/HL treatment.

#### PQ and tocopherol gene expression profile

We also quantified expression of two other genes, *hst1* and *mpbq-mt*, which encode proteins for the biosynthesis of PQ and tocopherol (Fig. 6A, red arrows). Levels of *hst*1 mRNA were up-regulated at 30 and 90 min of HL/HT treatment in the IL strain and at 15 min in the A250R mutant, with a weaker effect observed in S264K mutant after 90 min of treatment. Opposite to the latter, *mpbq-mt* mRNA was down-regulated at 15 min in all three lines, more severely so in the IL line. At 30 min, *mpbq-mt* mRNA levels were higher than the T0 point control while 90-min samples from IL and S264K lines had distinctly higher expression levels but not in the A250R line (Fig. 6B).

### Structural consequences of A250R and S264K substitutions in D1 protein

**Figure 7:**
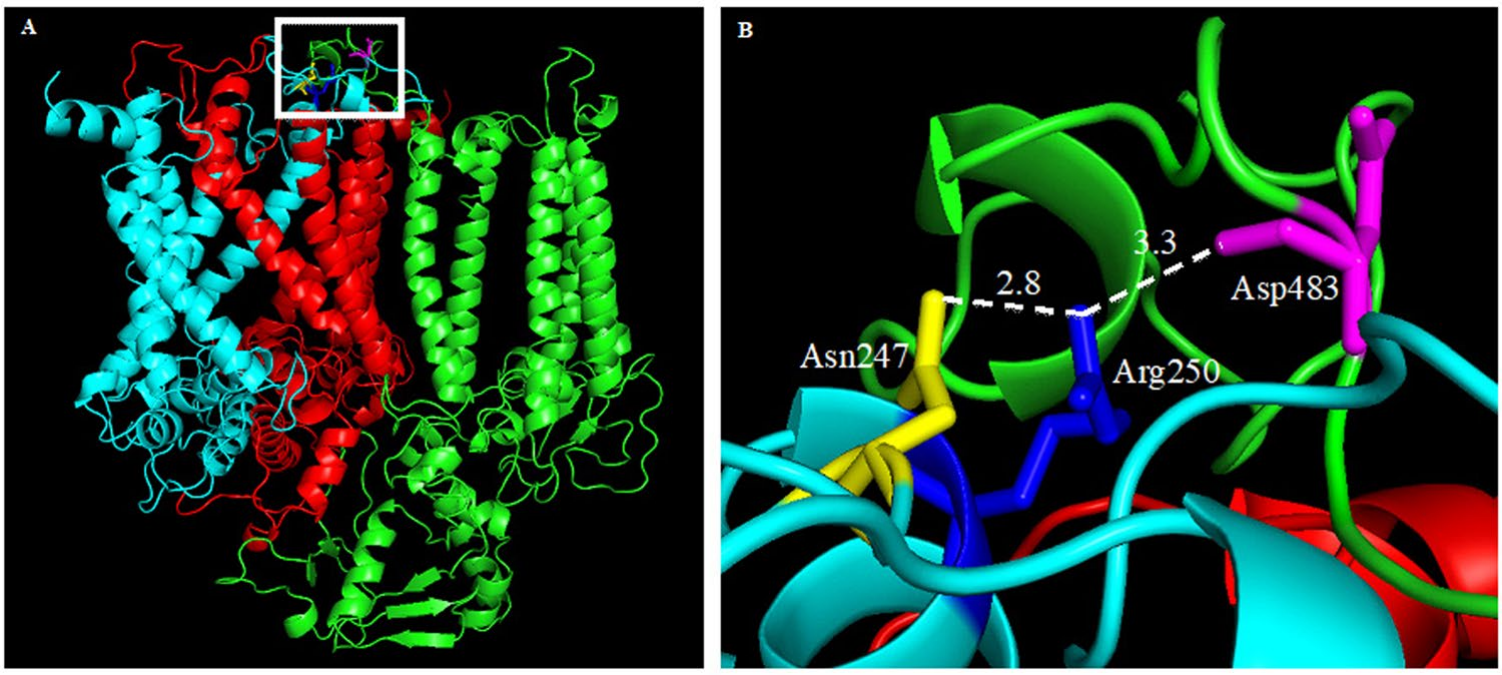
Environment of Arg250 in the A250R mutant. The positioning of Arg250 side chains was obtained by replacement of Ala250 in D1 protein structure using side chain modelling software^61^. (**A**) Cartoon presentation of D1 (light blue), D2 (red) and CP47 (green) proteins from the resolved structure of PSII (PDB, 3WU2). Residues Arg250 and Asn247 from D1, and residue Asp483 from CP47, are coloured in dark blue, yellow and magenta, respectively. Transmembrane helices of the proteins are clearly seen. Arg250 of the D1 protein is located in a short helix of the flexible loop between the D and E transmembrane helices as marked by the white square. (**B**) Details of the white square in A. Nearest atom distances are shown for two new hydrogen bonds formed upon mutation of Ala250 to Arg250. The pictures were created using the PyMol program.

The different effects on photochemistry and responses to HL/HT treatment of the D1 mutants in comparison to the control IL line prompted us to examine the structural consequences of A250R and S264K amino acid substitutions in the D1 protein by carrying out *in silico* analyses of the native and mutated Q_B_ binding pocket of *C*. *reinhardtii*. Residue Ala250 of the D1 protein is located in the short helix of the flexible loop between the D and E transmembrane helices (DE loop). In the resolved structure of PSII (PDB entry 3WU2), this residue sits at the interface of the D1 (chain A), D2 (chain D) and CP47 (chain B) proteins and does not directly interact with the plastoquinone (Q_B_) cofactor. Although the solvent accessible surface of the C beta atom of Ala250 is zero (the region is relatively dense), the possibility exists to mutate this residue to a more massive one with only small adjustments of side chains. Computational replacing of Ala with Arg at position 250 results in interatomic distances corresponding to the formation of two new hydrogen bonds, one to a side chain of Asn247 of the D1 protein and one to a side chain of Asp483 of the CP47 protein (Fig. 7). The two hydrogen bonds reduce flexibility in the DE loop. As a result, in the Arg250 mutant, the D1 protein is predicted to bind the Q_B_ cofactor but some chemical processes might be slowed due to the reduction in flexibility. On the other hand, the hydrogen bond added to CP47 could create a more stable PSII complex and might increase tolerance to various stress conditions.

**Figure 8:**
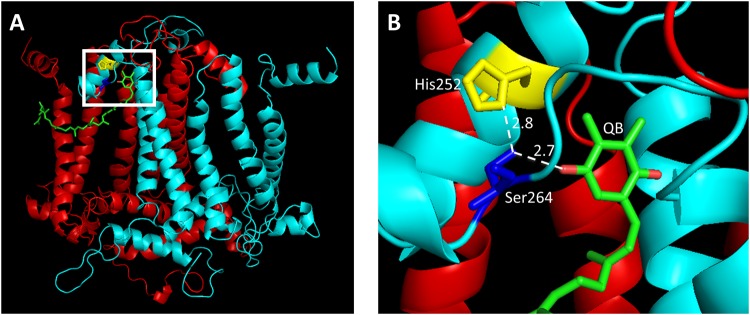
Environment of Ser264 in the D1 protein. Cartoons of the D1 (light blue) and D2 (red) proteins are presented. Ser264 and His252 are coloured dark blue and yellow, respectively. The QB molecule is coloured by atoms (carbon = green, oxygen = red). (**A**) The overall structure of the D1/D2 heterodimer proteins. The region of D1 protein residue Ser264 is located at the membrane/cytosol boundary and is marked by a white square. (**B**) Details of the white square in A. Nearest atom distances for the two H-bonds discussed in the text are shown. The picture was created using the resolved structure of PSII (PDB, 3WU2) and using the PyMol program.

Residue Ser264 of the D1 protein sits in the flexible DE loop (Fig. 8). This residue is located at the membrane boundary and forms hydrogen bonds with Q_B_ and His252 of the D1 protein^6^. Thus, Ser264 plays important roles both in plastoquinone binding and in stabilizing the binding pocket^35^. CSU analysis^36^ shows that both the carbon beta and oxygen gamma atoms of Ser264 have solvent accessible surfaces equal to zero indicating that the side chain of the residue is pointing into a densely crowded region. This, along with visual inspection, suggest that when complexed with Q_B_, there is no space in the D1 structure for replacement of Ser264 with a considerably larger lysine residue. In the apo form of the mutated protein (which lacks the quinone cofactor), there is space for Lys264. However, in this case, complex formation with Q_B_ would require extensive structural rearrangement of the binding region. We speculate that formation of such a complex will be more difficult (if possible) and could adversely affect growth. This finding is in-line with our previous results demonstrating that the PQ pool reduction rate is highly delayed in the *Chlamydomonas* D1 S264K mutant, having rate constants similar to those measured in an atrazine-treated IL line^37^.

## Discussion

We demonstrate here that amino acid substitutions A250R and S264K in the PSII D1 protein remodel H-bond network at Q_B_ site of D1, affect the photosynthetic efficiency, and metabolically re-wire *C*. *reinhardtii* under normal physiological conditions. Mutation at any point which would negatively affect the electron transfer in PSII, either on the level of the D1 protein or another essential protein, could be crucial and would have probably a similar effect on PSII susceptibility to photoinhibition and changes in the pigment composition or metabolic content. Exposure to HL/HT stress accentuated metabolic consequences in the mutants and the parental IL line. Both mutations impeded the reoxidation rate of the primary electron acceptor quinone Q_A_ and oxygen production capacity without a severe effect on maximum quantum yield of PSII photochemistry in normal conditions. The relevance of the D1-Ser264 residue in maintaining a normal electron transfer between Q_A_ and Q_B_ is apparent by 50% reduction in *1-V*_*J*_ and delay in reaching the maximum fluorescence level in the mutant compared to the parent IL (Fig. 1, Table S1). This also indicates a hindered reduction either of the Q_B_ docked in the D1 binding site or of the PQ pool reduction rate in the S264K mutant^37^. Moreover, we demonstrate that the S264K and A250R mutants are impacted in the content of violaxanthin, antheraxanthin, lutein and β-carotene, but not zeaxanthin, the accessory pigments that promote light-harvesting and/or photoprotection^38,39^. Notably, the differences in photosynthetic parameters, including reduced content of accessory pigments and Chl*a* cell^−1^ suggest that the two D1 mutants behave as organisms adapted to excessive light energy – with a possible reduced antenna cross-section conferring an acclimation advantage by lowering the *elicitation sensing* due to a lower number of captured photons^40,41^ (Figs S2 and S5).

The targeted metabolomics provided a window into alterations in the accumulation patterns of specific metabolites (and therefore pathways) in A250R and S264K compared to the control IL (Fig. 5, Table S2). For instance, Phe and Tyr are precursors for the synthesis of plant pigments, including the carotenoids and PQ, respectively, via coumarate and acetoacetyl CoA. Both these metabolites were enriched in both mutants than the parental line. Tyr is a precursor of fumarate and both accumulate in the mutants compared to the parental line. Fumarate is a precursor of Asp, which can feed into the Arg/Orn pathway and produce a stress indicator as diamine putrescine, which is also upregulated in the mutants. Lys (produced from Asp) is also enriched in both the two mutants, and its metabolism leads to acetoacetyl CoA that provides precursors for carotenoids and the xanthophyll cycle via the mevalonate pathway. Malate and succinate accumulation can influence the NAD^+^/NADPH(H) ratio, involving tricarboxylic acid and Fe-S cycling. In addition, fumarate, succinate, AMP, Thr and NAD^+^ are equally relevant for Chl and carotenoid biogenesis. Moreover, since tricarboxylic acid cycle involves both malate and acetyl COA one may also predict that D1 mutation somehow communicates with the respiratory organelle mitochondria. Thus, the sustenance of photochemistry associated with specific changes in metabolism including pigment biosynthesis links the two D1 mutations to metabolic alterations. In this connection, a pronounced modulation of these metabolites was apparent upon HL/HT stress of the two mutants and the parent IL. HL/HT stress increased the total content of pigments involved in the xanthophyll cycle in IL and A250R (~15-fold and ~4-fold, respectively), thus highlighting a robust stress-response by activating the xanthophyll cycle causing accumulation of zeaxanthin (Fig. 4). Similarly, β-carotene accumulation within 90 min of HT/HL stress can be interpreted as a means to increase radical-scavenging capacity and/or as a substrate for the *de novo* zeaxanthin synthesis^38^. Interestingly, such an increase was not observed with the S264K mutant, suggesting that this mutation prevents the ability to cope with and sustain the double-stress conditions.

In congruence with the metabolomics data discussed above, upregulation of the biosynthesis genes for the carotenoid pathway, in particular zeaxanthin and lutein, over that of the PQ/tocopherol biosynthetic branch, demonstrates ‘gene to metabolite’ communication as an early robust response of *Chlamydomonas* to environmental changes, here involving cues generated from studying these specific D1 mutants. Thus, early induction of *psy* (only in IL and A250R) and *chy-β* (all strains) transcripts occurs in response to stress. Also of note is that once zeaxanthin and lutein accumulated, the levels of *chy-β* transcripts decreased (see data at 90 min of exposure) (Fig. 6). A similar scenario was also apparent between β-carotene accumulation and *lyc-β* transcripts, suggesting also of a negative feedback regulation to downregulate gene expression as and when needed. With longer duration of stress, induction of the *mpbq-mt* gene, involved in the PQ/tocopherol biosynthetic branch was apparent. The late activation of this pathway could fulfil the requirement for *de novo* synthesis of the electron acceptor PQ in the thylakoids, and/or of intermediate precursors in tocopherol synthesis. This speculation remains to be confirmed, however, it is noted that PQ has an essential role also as a hydrogen acceptor in the desaturation reactions by phytoene desaturase and ζ-carotene desaturase, involved in the initial steps of the carotenoid biosynthetic pathway^38,42^. Considering that carotenoid and PQ biosynthesis share geranylgeranyl diphosphate as a common precursor, the early phase of stress response seems to create first the need for carotenoids and thereafter for PQ. These observations are in line with the findings that PQ over-reduction leads to thylakoid lumen acidification, which is considered as an important signal for inducing the xanthophyll cycle-dependent dissipation of excess energy^43,44^. Likewise, tocopherols localized in the thylakoids maintain membrane stability and scavenge any singlet oxygen produced during PSII reaction centre function. Previously, it was shown that chloroplast tocopherols are light-modulated and triggered by the increased P680 triplet state at HL intensity^45^.

Both the mutants, A250R and S264K, were found to have specific but different transcript profiles compared to IL. The single amino acid substitutions in them are resident at the Q_B_ binding niche, however, in the case of the S264K mutant, the replaced amino acid is directly involved in the interaction between the D1 protein and Q_B_ molecule^37,46^, forming a new hydrogen bond. Our results suggest that modifications at these sites may restrict or prevent Q_B_ binding and, therefore, impact electron flow rate from PSII to PSI under both normal and stress conditions. This could generate different and/or delayed signals within the cell. In this context, the *in silico* analyses concerning the interactions between functional groups of A250 and S264 and the D1 protein residues in the neighbouring environment strongly support the novel features of mutants. In particular, our analysis indicates that the A250R mutation does not negatively affect the protein structure; on the contrary, S264K substitution generates a less stable protein. Thus, the *in silico* analysis supports our findings of similar photosynthetic performance of A250R and parent IL strain, which is impaired in the S264K mutant.

In the natural environment, the occurrence of spatial and temporal gradients of light quality, light intensity, temperature and nutrient levels trigger intracellular signalling pathways that control both the biogenesis and performance of the photosynthetic apparatus through short- and long-term adaptive responses^47–49^. In this context, the chloroplast behaves as a sensor and transducer of environmental cues to communicate with the other intracellular compartments. This mechanism, known as retrograde signalling, involves modification of reactive oxygen species levels, metabolites, hormones and redox homeostasis that convey messages to the cytoplasmic organelles to remodel gene expression, translational and post-translation events^50,51^. The redox state of the photosynthetic electron transport chain is considered one of the main control points regulating retrograde signalling. Plants and green algae impaired in the redox poise control are also impaired in efficient signalling systems^52,53^. Based on the latter observations and our studies presented here, it is tempting to speculate that the dynamic D1 protein may be a site in the chloroplast for generating signals involved in retrograde pathways.

## Materials and Methods

### Strains and growth conditions

Single point mutants were produced by particle gun transformation of Del1 strain^54^ and the control was *C*. *reinhardtii* IL strain^55^. The amino acid substitutions of Ala250 with Arg (A250R) and Ser264 with Lys (S264K) were introduced by site directed mutagenesis^14^ using specific primer pairs (Table S4). All strains were grown in Tris-acetate-phosphate medium (pH 7.2)^56^ at 50 µmol m^−2^ s^−1^ and 25 °C before transfer to other specified experimental conditions. Cell growth (culture density, OD) and total Chl content were determined spectrophotometrically at 750 nm and 652 nm, respectively^57^. Antenna size as µg Chl*a* per cell^39^ and Chl*a*^58^ content were determined. For cell count, a Thoma chamber was used (0.100 mm Tiefe Depth, 0.0025 mm^2^ area). The cell size distribution was quantified after adding 0.1 ml of Lugol’s iodine solution to 0.9 ml of *C*. *reinhardtii* cultures. The images were processed and analyzed using ImageJ software^59^. The set scale was calibrated as 0.86 pixels/µm and the software approximated the cells as ellipses with the primary and secondary axes considered as major and minor diameters, respectively. For each strain, over 100 cells were counted. All experiments were performed in quadruplicate for each strain, using cultures in the early exponential growth phase (OD < 0.4).

### Fluorescence measurements

The *OJIP* fluorescence transients were registered by a Plant Efficiency Analyzer (Hansatech Instr. Ltd, Kings Lynn, Norfolk, UK) in liquid cell cultures^14^. Maximum quantum yield of PSII photochemistry was calculated as *F*_*v*_*/F*_*m*_ = (*F*_*m*_ − *F*_0_)*/F*_*m*_, where *F*_*v*_ is the variable fluorescence, *F*_0_ is the initial fluorescence level registered at 50 µs after the onset of illumination, and *F*_*m*_ is the maximum fluorescence. The relative variable fluorescence curves (*Vt*) were calculated as *V*_*t*_ = (*F*_*t*_ − *F*_0_)*/*(*F*_*m*_ − *F*_0_). The electron transport efficiency between Q_A_ and Q_B_ quinones of PSII was estimated by the parameter *1* − *V*_*J*_ = *1-*(*F*_*J*_ − *F*_0_)*/*(*F*_*m*_ − *F*_0_), where *F*_*J*_ represents the fluorescence level at 2 ms after the onset of illumination^34^.

### Oxygen evolution analyses

Cells photosynthetic activity was measured at 24 °C using a Clark-type oxygen electrode (Chlorolab 2 System, Hansatech Instr. Ltd, Kings Lynn, Norfolk, UK) as previously reported^14^. Each sample in TAP medium (pH 7.2) contained 1-ml of algal cell culture (18 ± 2 µg ml^−1^ total chlorophyll) and 10 mM NaHCO_3_ as an additional carbon source^60^. Light-saturation curves of oxygen evolution were obtained using a red LED light source (with maximum at 650 nm), starting with registration of the dark respiration, followed by measurement of the oxygen production rate at 10, 30, 50, 80, 100, 200, 300 and 350 µmol m^−2^ s^−1^. At each light intensity, the rate of oxygen evolution was recorded continuously for 2 min. Photosynthetic efficiency of each line was calculated by regression analyses of the linear part of the light-saturated curve, and the light compensation point as the light intensity at which the photosynthetic O_2_ evolution reached equilibrium with the respiratory O_2_ uptake^61^.

### Computational analyses

Side chain placement of the mutated residue in each mutant was performed using SCCOMP software with an option to optimize positions of first-sphere residues^62^. Analysis of changes upon protein mutation was performed by LPC/CSU software^36^. Molecular graphics were generated with the PyMOL Molecular Graphics System (DeLano Scientific LLC, Palo Alto, CA, USA; http://www.pymol.org).

### HPLC analysis and quantification of pigment levels

All chemicals and solvents were HPLC grade. All the standards were prepared under dim (or green safe) light at 4 °C. Nitrogen gas was flushed into the vials to avoid degradation and isomerization of pigments. All the vials were stored at −20 °C in the dark. The HPLC column was an YMC C30 column (Waters, USA), 5 µm, 250 × 4.6 mm, protected by a C18 (1 cm) guard column and in-line filter (0.22 µm). Working standard solutions were diluted with methanol-acetonitrile-water (84:14:2, v/v/v) mixture (solvent A) to avoid solvent interference. The HPLC calibration curve of each standard was analysed at least three times. Equal volumes of algal culture were harvested from each strain and the cell number determined as described above. Cells were then harvested by centrifugation (15 min, 1500 g, 4 °C), and the pellets immediately frozen using liquid N_2_ and lyophilized overnight. The lyophilized samples were re-hydrated by adding 200 µl of HPLC grade water, supplemented with Na_2_CO_3_ crystals (to maintain pH during cells lysis), and then re-suspended in 100% cold acetone. The suspension was hence centrifuged (1500 g, 15 min, 4 °C) recovering the supernatant. This extraction procedure was repeated until the pellet bleached, then the supernatants were combined. Before HPLC injection, the extracts were filtered through a 0.45 µm PVDF filter and dried under N_2_. Powders were solubilised with 400 µl of solvent A and stored at −20 °C until use. Each analysis was carried by injecting a 20 µl sample and repeated three times. When necessary, standards were added to the sample extract for co-chromatography. The mobile phase system was comprised of a mixture of solvent A and 100% methylene chloride (solvent B). The column temperature was maintained at 21 °C.

### NMR analysis of targeted metabolites

Nuclear Magnetic Resonance spectroscopy was used to quantify levels of targeted metabolites. Harvesting and preparation of *Chlamydomonas* pellets were carried out as described above for HPLC analysis. Lyophilized pellets were finely powdered and extracted with methanol/chloroform/water in 2:2:1 volumetric ratio^63^. After centrifugation (11000 g, 20 min, 4 °C), the upper hydro-alcoholic phase and the lower organic phase were carefully collected and dried under N_2_. The water-soluble extract was solubilized in 0.75 mL D_2_O phosphate buffer (400 mM, pH 7.0) with 1 mM sodium trimethylsilylpropionate-2,2,3,3-d_4_ (TSP) as an internal standard. The NMR spectra were recorded at 27 °C on a Bruker AVANCE 600 NMR spectrometer operating at the proton frequency of 600.13 MHz. ^1^H spectra were referenced to the methyl group signal of TSP (δ = 0.00 ppm) in D_2_O. The acquisition parameters of the ^1^H spectra were as follows: 256 scans, recycle delay of 7 s, spectral width 7.2 kHz, time domain 32768. The residual HDO signal was suppressed using pre-saturation by a soft pulse during 2 s just before acquisition. For the assignment of ^1^H NMR spectra, a set of 2D NMR experiments (^1^H-^1^H TOCSY, ^1^H-^13^C HSQC, ^1^H-^13^C HMBC) was performed as previously decribed^64^. The spin lock field for the ^1^H-^1^H TOCSY was 6250 Hz, and mixing time was 80 ms. The HSQC experiment was performed using a coupling constant ^1^*J*_C-H_ of 150 Hz, and the delay for the evolution of long-range couplings in the ^1^H-^13^C HMBC experiment was 80 ms. Spectral processing was carried out using Bruker Topsin 1.3 software. After Fourier transformation, manual phase and baseline corrections ^1^H spectra were divided into small regions of 0.02-0.03 ppm each and integrated. The total sum of 139 integrals was normalized to 1000. The regions of HDO and Tris signals (at 4.80 and 3.75 ppm, respectively) were excluded from the normalization and statistical analysis. After normalization of the total sum of integrals to a constant value, all the spectra were normalized using probabilistic quotient normalization (PQN)^65^. This method is based on the calculation of the most probable dilution factor by looking at the distribution of the quotients obtained by dividing the integrals of the acquired spectra by those of a reference spectrum. The median spectrum of the control samples was used as reference.

### Total RNA extraction and quantitative real-time PCR (qRT-PCR)

Cell cultures (OD_750_ = 0.40) corresponding to 2.2 × 10^6^ cells ml^−1^ for IL, 5.5 × 10^6^ cells ml^−1^ for A250R and 6.4 × 10^6^ cells ml^−1^ for S264K, were harvested and total RNA extracted as previously described^66^. Each RNA sample was subjected to DNase treatment using a Turbo DNA-free kit (Ambion). Total RNA (80 ng) was reverse transcribed and amplified using the SYBR Green PCR Master Mix and MuLV Reverse Transcriptase Reagents, according to one-step RT-PCR manufacturer’s protocol (Applied Biosystems, Foster city, USA). qRT-PCRs were performed in the Applied Biosystems 7900HT Real Time System with a standard 96-Well Block Module, using Frosted Subskirted optical tubes and Seal Film (Applied Biosystems, Foster city, USA). The reactions were subjected to a heat dissociation protocol using the 7900HT System software for melting curve analysis and detection of non-specific amplifications. A negative control without template was run with each assay to assess the overall specificity. The relative abundance of each gene was determined by the 2^−∆∆Ct^ method^67^. RACK1 (receptor of activated protein kinase C1)^68^ was used as the endogenous control and for determining relative abundance of transcripts. Each assay included triplicate reactions. Primers were designed using Primer Quest (Integrated DNA Technologies, Coralville) (Table S5).

### Statistical analyses

Data presented are means of three independent experiments, each with three technical repetitions. The differences between the parent strain and the two D1 mutants were assessed by a non-parametric Mann-Whitney U test for comparing independent samples. The statistical significance of the differences was evaluated by p-values ≤ 0.05. The NMR data were subjected to PCA and ANOVA analyses using the STATISTICA package for Windows. Before the PCA analysis, the data were mean-centred and each variable was divided by its standard deviation (autoscaling).

## Electronic supplementary material

Supplementary File

Dataset 1

Dataset 2

Dataset 3

Dataset 4

## Data Availability

The datasets generated during and/or analysed during the current study are included in this published article (and its Supplementary Information files).

## References

[1] Barber J (2006). Photosystem II: an enzyme of global significance. Biochem. Soc. Transac..

[2] Blankenship RE (2010). Early evolution of photosynthesis. Plant Physiol..

[3] Mattoo AK, Hoffman-Falk H, Marder JB, Edelman M (1984). Regulation of protein metabolism: Coupling of photosynthetic electron transport to *in vivo* degradation of the rapidly-metabolized 32-kilodalton protein of the chloroplast membranes. Proc. Natl. Acad. Sci., USA..

[4] Yokthongwattana, K. & Melis, A. Photoprotection, Photoinhibition, Gene Regulation, and Environment. Advances in *Photosynthesis and Respiration* (eds Demmig-Adams, B., Adams, W. & Mattoo, A.) 175–191 (Springer, 2006).

[5] Edelman M, Mattoo AK (2008). D1-protein dynamics in photosystem II: the lingering enigma. Photosynth. Res..

[6] Ferreira KN, Iverson TM, Maghlaoui K, Barber J, Iwata S (2004). Architecture of the photosynthetic oxygen-evolving center. Science.

[7] Loll B, Kern J, Saenger W, Zouni A, Biesiadka J (2005). Towards complete cofactor arrangement in the 3.0 Å resolution structure of photosystem II. Nature.

[8] Guskov A (2009). Cyanobacterial photosystem II at 2.9-A resolution and the role of quinones, lipids, channels and chloride. Nat. Struct. Mol. Biol..

[9] Umena Y, Kawakami K, Shen JR, Kamiya N (2011). Crystal structure of oxygen-evolving photosystem II at a resolution of 1.9Å. Nature.

[10] Suga M (2014). Native structure of photosystem II at 1.95 Å resolution viewed by femtosecond X-ray pulses. Nature.

[11] Tanaka A, Fukushima Y, Kamiya N (2017). Two different structures of the oxygen-evolving complex in the same polypeptide frameworks of photosystem II. J. Am. Chem. Soc..

[12] Rea G (2009). Structure‐based design of novel *Chlamydomonas reinhardtii* D1‐D2 photosynthetic proteins for herbicide monitoring. Protein Sci..

[13] Rea, G. *et al*. Computational biology, protein engineering, and biosensor technology: a close cooperation for herbicides monitoring. In *Herbicides*, *Theory an*d Applica*ti*on*s* (eds Soloneski, S. & Larramendy, M. L.) 93–120 (InTech, 2011).

[14] Rea G (2011). Directed evolution and *in silico* analysis of reaction centre proteins reveal molecular signatures of photosynthesis adaptation to radiation pressure. PLoS One.

[15] Lambreva MD (2013). A powerful molecular engineering tool provided efficient *Chlamydomonas* mutants as bio-sensing elements for herbicides detection. PloS One.

[16] Hirschberg J, McIntosh L (1983). Molecular basis of herbicide resistance in *Amaranthus hybridus*. Science.

[17] Goloubinoff P, Edelman M, Hallick RB (1984). Chloroplast-coded atrazine resistance in *Solanum nigrum*: psbA loci from susceptible and resistant biotypes are isogenic except for a single codon change. Nucl. Acid Res..

[18] Ohad N, Hirschberg J (1992). Mutations in the D1 subunit of photosystem II distinguish between quinone and herbicide binding sites. Plant Cell.

[19] Mattoo AK, Marder JB, Gressel J, Edelman M (1982). Presence of the rapidly-labeled 32,000-dalton chloroplast membrane protein in triazine resistant biotypes. FEBS Lett..

[20] Lardans A (1998). Biophysical, biochemical, and physiological characterization of *Chlamydomonas reinhardtii* mutants with amino acid substitutions at the Ala251 residue in the D1 protein that result in varying levels of photosynthetic competence. J. Biol. Chem..

[21] Rose S (2008). D1-arginine257 mutants (R257E, K, and Q) of *Chlamydomonas reinhardtii* have a lowered QB redox potential: analysis of thermoluminescence and fluorescence measurements. Photosynth. Res..

[22] Sundby C, Soon Chow W, Anderson JM (1993). Effects on photosystem II function, photoinhibition, and plant performance of the spontaneous mutation of Serine-264 in the Photosystem II reaction center D1 protein in triazine-resistant *Brassica napus* L. Plant Physiol..

[23] Perewoska I, Etienne AL, Miranda T, Kirilovsky D (1994). Destabilization and higher sensitivity to light in metribuzin-resistant mutants. Plant Physiol..

[24] Bajkán S (2010). Conserved structure of the chloroplast-DNA encoded D1 protein is essential for effective photoprotection via non-photochemical thermal dissipation in higher plants. Mol. Genet. Genomics.

[25] Przibilla E, Heiss S, Johanningmeier U, Trebst A (1991). Site-specific mutagenesis of the D1 subunit of photosystem II in wild-type Chlamydomonas. Plant Cell.

[26] Alfonso M (2001). Unusual tolerance to high temperatures in a new herbicide-resistant D1 mutant from *Glycine max* (L.) Merr. cell cultures deficient in fatty acid desaturation. Planta.

[27] Roncel M (2007). Changes in photosynthetic electron transfer and state transitions in an herbicide-resistant D1 mutant from soybean cell cultures. Biochim. Biophys. Acta.

[28] Mattoo AK, St John JB, Wergin WP (1984). Adaptive reorganization of protein and lipid components in chloroplast membranes as associated with herbicide binding. J. Cell. Biochem..

[29] Shlyk-Kerner O (2006). Protein flexibility acclimatizes photosynthetic energy conversion to the ambient temperature. Nature.

[30] Shlyk O (2017). A single residue controls electron transfer gating in photosynthetic reaction centers. Sci. Rep..

[31] Summerfield TC, Toepel J, Sherman LA (2008). Low-oxygen induction of normally cryptic psbA genes in cyanobacteria. Biochem..

[32] Yoshioka M (2006). Quality control of photosystem II cleavage of reaction center D1 protein in spinach thylakoids by FtsH protease under moderate heat stress. J.Biol.Chem..

[33] Johanningmeier U (2000). Herbicide resistance and supersensitivity in Ala250 or Ala251 mutants of the D1 protein in *Chlamydomonas reinhardtii*. Pest. Biochem. Physiol..

[34] Strasser, R.J., Srivastava, A. & Tsimilli-Michael, M. The fluorescence transient as a tool to characterize and screen photosynthetic samples. *In Probing Photosynthesis: Mechanism*, *Regulation and Adaptation* (eds Yunus, M., Pathre, U. & Mohanty, P.) 443–480 (Taylor and Francis, London, 2000).

[35] Sobolev V, Edelman M (1995). Modeling the quinone-B binding site of the photosystem-II reaction center using notions of complementarity and contact surface between atoms. Proteins.

[36] Sobolev V, Sorokine A, Prilusky J, Abola EE, Edelman M (1999). Automated analysis of interatomic contacts in proteins. Bioinformatics.

[37] Zobnina V (2017). The plastoquinol–plastoquinone exchange mechanism in photosystem II: insight from molecular dynamics simulations. Photosyn. Res..

[38] Grossman AR, Lohr M, Im CS (2004). *Chlamydomonas reinhardtii* in the landscape of pigments. Annu. Rev. Genet..

[39] Nisar N, Li L, Lu S, Khin NC, Pogson BJ (2015). Carotenoid metabolism in plants. Mol. Plant.

[40] Laroche J, Mortain-Bertrand A, Falkowski PG (1991). Light intensity-induced changes in cab mRNA and light harvesting complex II apoprotein levels in the unicellular chlorophyte *Dunaliella tertiolecta*. Plant Physiol..

[41] Polle JWE, Niyogi KK, Melis A (2001). Absence of lutein, violaxanthin and neoxanthin affects the functional chlorophyll antenna size of Photosystem-II but not that of Photosystem-I in the green alga *Chlamydomonas reinhardtii*. Plant Cell Physiol..

[42] Norris SR, Barrette TR, DellaPenna D (1995). Genetic dissection of carotenoid synthesis in Arabidopsis defines plastoquinone as an essential component of phytoene desaturation. Plant Cell.

[43] Wilson KE, Huner NP (2000). The role of growth rate, redox-state of the plastoquinone pool and the trans-thylakoid delta pH in photoacclimation of *Chlorella vulgaris* to growth irradiance and temperature. Planta.

[44] Woitsch S, Römer S (2003). Expression of xanthophyll biosynthetic genes during light-dependent chloroplast differentiation. Plant Physiol..

[45] Trebst A, Depka B, Holländer-Czytko H (2002). A specific role for tocopherol and of chemical singlet oxygen quenchers in the maintenance of photosystem II structure and function in *Chlamydomonas reinhardtii*. FEBS Lett..

[46] Saito K, Rutherford AW, Ishikita H (2013). Mechanism of proton coupled quinone reduction in Photosystem II. Proc. Natl. Acad. Sci. USA.

[47] Eberhard S, Finazzi G, Wollman FA (2008). The dynamics of photosynthesis. Annu. Rev. Genet..

[48] Kono M, Terashima I (2014). Long-term and short-term responses of the photosynthetic electron transport to fluctuating light. J. Photochem. Photobiol. B Biol..

[49] Rochaix Jean-David (2016). The Dynamics of the Photosynthetic Apparatus in Algae. Applied Photosynthesis - New Progress.

[50] Chan KX, Phua SY, Crisp P, McQuinn R, Pogson BJ (2016). Learning the languages of the chloroplast: retrograde signaling and beyond. Annu. Rev. Plant Biol..

[51] de Souza A, Wang JZ, Dehesh K (2017). Retrograde signals: integrators of interorganellar communication and orchestrators of plant development. Annu. Rev.Plant Biol..

[52] Tikkanen M., Gollan P. J., Mekala N. R., Isojarvi J., Aro E.-M. (2014). Light-harvesting mutants show differential gene expression upon shift to high light as a consequence of photosynthetic redox and reactive oxygen species metabolism. Philosophical Transactions of the Royal Society B: Biological Sciences.

[53] Rea Giuseppina, Antonacci Amina, Lambreva Maya D., Mattoo Autar K. (2018). Features of cues and processes during chloroplast-mediated retrograde signaling in the alga Chlamydomonas. Plant Science.

[54] Preiss S, Schrader S, Johanningmeier U (2001). Rapid, ATP-dependent degradation of a truncated D1 protein in the chloroplast. Eur. J. Biochem..

[55] Johanningmeier U, Heiss S (1993). Construction of a *Chlamydomonas reinhardtii* mutant with an intronless *psb*A gene. Plant Mol. Biol..

[56] Harris, E. *The Chlamydomonas Source-Book*. (Academic Press, New York, 1989).

[57] Walker, D. A. Preparation of higher plant chloroplasts. *In Methods Enzymol*., Vol. 69, (ed. San Pietro A.) 94–104 (Academic Press, New York, 1980).

[58] Lichtenthaler HK (1987). Chlorophylls and carotenoids: pigments of photosynthetic biomembranes. Methods Enzymol..

[59] Rasband, W.S. ImageJ, U.S. National Institute of Health, Bethesda, Maryland, USA, http://rsb.info.nih.gov/ij/, 1997–2007.

[60] Melis A, Neidhardt J, Benemann JR (1999). *Dunaliella salina* (Chlorophyta) with small chlorophyll antenna sizes exhibit higher photosynthetic productivities and photon use efficiencies than normally pigmented cells. J. Appl. Phycol..

[61] Walker, D. The use of the oxygen electrode and fluorescence probes in simple measurements of photosynthesis http://citeseerx.ist.psu.edu/viewdoc/download?doi=10.1.1.129.2517&rep=rep1&type=pdf (1990).

[62] Eyal E, Najmanovich R, McConkey BJ, Edelman M, Sobolev V (2004). Importance of solvent accessibility and contact surfaces in modeling side-chain conformations in proteins. J. Comput. Chem..

[63] Mannina L, Sobolev AP, Capitani D (2012). Applications of NMR metabolomics to the study of foodstuffs: Truffle, kiwifruit, lettuce, and sea bass. Electrophoresis.

[64] Mannina L, Cristinzio M, Sobolev AP, Ragni P, Segre A (2004). High-field nuclear magnetic resonance (NMR) study of truffles (Tuber *Aestivum vittadini*). J. Agric. Fd. Chem..

[65] Dieterle F, Ross A, Schlotterbeck G, Senn H (2006). Probabilistic quotient normalization as robust method to account for dilution of complex biological mixtures. Application in ^1^H NMR metabonomics. Anal. Chem..

[66] Lake V, Willows RD (2003). Rapid extraction of RNA and analysis of transcript levels in Chlamydomonas reinhardtii using real-time RT-PCR: magnesium chelatase *chlH*, *chlD* and *chlI* gene expression. Photosynth. Res..

[67] Livak KJ, Schmittgen TD (2001). Analysis of relative gene expression data using real-time quantitative PCR and the 2(-Delta Delta C(T)) method. Methods.

[68] Schloss JA (1990). A Chlamydomonas gene encodes a G protein beta subunit-like polypeptide. Mol. Gen. Genet..

