# Supplementary material for: Photosystem-II D1 protein mutants of Chlamydomonas reinhardtii in relation to metabolic rewiring and remodelling of H-bond network at QB site
Source: Sci Rep. 2018 Oct 3;8:14745. doi: 10.1038/s41598-018-33146-y (PMC6170454; doi:10.1038/s41598-018-33146-y)

**Supplementary Information**

**Photosystem-II D1 protein mutants of *Chlamydomonas reinhardtii* in relation to metabolic rewiring and remodelling of H-bond network at QB site**

**Amina Antonacci^1^, Maya D. Lambreva^1^, Andrea Margonelli^1^_,_ Anatoly Sobolev^2^, Sandro Pastorelli^1^, Ivo Bertalan^3^, Udo Johanningmeier^3^, Vladimir Sobolev^4^, Ilan Samish^4,5^, Marvin Edelman^4^, Vesa Havurinne^7^, Esa Tyystjärvi^7^, Maria T. Giardi^1^, Autar K. Mattoo^6*^, and Giuseppina Rea^1*^**

# ^1^Institute of Crystallography, National Research Council of Italy, Via Salaria Km 29,3 00015 Monterotondo Stazione, Rome, Italy

^2^Institute of Chemical Methodologies, National Research Council of Italy, Via Salaria km 29,3 00015 Monterotondo Stazione, Rome, Italy
^3^Current address: Neotron S.p.a., Santa Maria di Mugnano, Modena, Italy

# ^4^Martin-Luther-University, Plant Physiology Institute, Weinbergweg 10 D-06120 Halle (Saale), Germany

^5^Department of Plant and Environmental Sciences, Weizmann Institute of Science, Rehovot, Israel
^6^Current address: Amai Proteins Ltd., 2 Bergman St. Rehovot, Israel

^7^Department of Biochemistry/Molecular Plant Biology, FI-20014, University of Turku, Turku, Finland

^8^The Henry A Wallace Beltsville Agricultural Research Centre, United States Department of Agriculture, Sustainable Agricultural Systems Laboratory, Beltsville, Maryland 20705, USA

*To whom correspondence should be addressed:

[giuseppina.rea@ic.cnr.it](mailto:giuseppina.rea@ic.cnr.it); telephone: +390690672631 fax: +390690672630

Autar.Mattoo@ars.usda.gov; telephone: +1 3015046622; fax +1 301 5046492

**Figures and Tables are organized as they appear in the running text.**

**Short Legend**

**Figure S1**. The growth rate of different *C. reinhardtii* IL, A250R and S264K strains.

**Figure S2.** Accumulation levels of carotenoids in *C. reinhardtii* IL, A250R and S264K strains under physiological conditions.

**Figure S3.** An expanded PCA scores plot region where A250R and S264K sample scores are located.

**Figure S4.** Correlation plots for b-carotene/a-carotene (A) and chlorophylls *a* and *b* (B).

**Figure S5**. Chl*a* content per cell in the *C. reinhardtii* strains.

**Figure S6.** Schematic representation of the *C. reinhardtii* strains response to HL/HT**.**

**Table S1.** Photosynthetic parameters of IL parent strain and D1 site-directed mutants of *C. reinhardtii*.

**Table S2.** NMR signal assignment and quantification of metabolite levels.

**Table S3.** PCA analysis reporting ratios (A250R/S264K) of mean values of pigments.

**Table S4.** Primer pairs used in the two-step PCR for the site-directed mutagenesis.

**Table S5.** Sequences of primers used in the qRT-PCR experiments.

**Figure S1.** The time courses of cell culture growth A) and chlorophylls accumulation B) of *C. reinhardtii* D1 strains during a period of 168 h. The difference between the mutants and the parent strain gradually increased with time. At least three separate repetitions were done per each strain. Each point represents the average of three experiments ± SD. P≤0.05 (Mann-Whitney U Test).


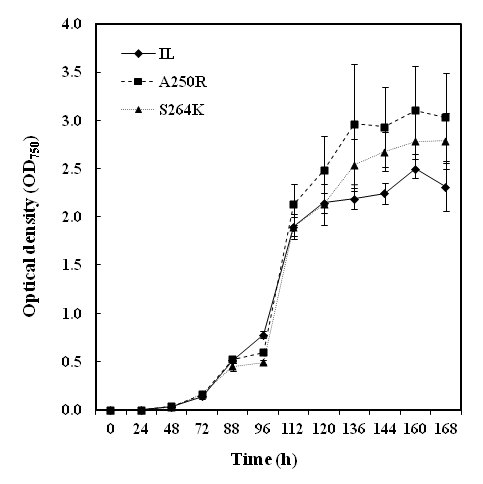

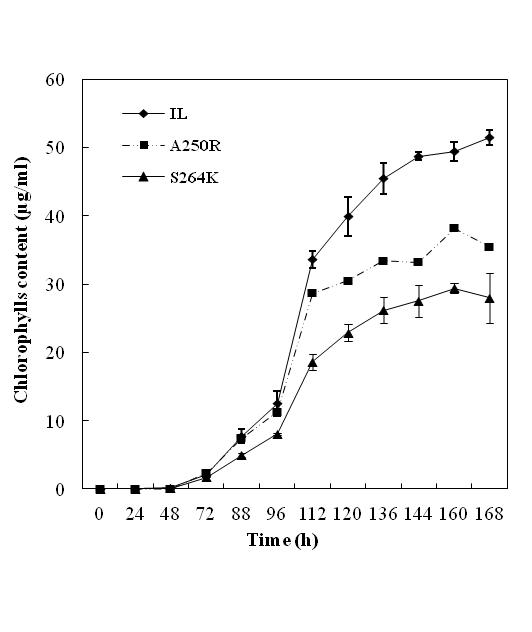


**A**

**B**

**Table S1.** Photosynthetic parameters of IL parent strain and D1 site-directed mutants of *C. reinhardtii*. All parameters were determined in cell cultures in early-exponential growth phase, corresponding to OD_750_=0.35±0.05. The maximum quantum yield of PSII photochemical reaction (*F_v_/F_m_=(F_m_-F_0_)/F_m_*) and the PSII electron transport efficiency (*1-V_J_=(1-(F_J_-F_0_)/(F_m_-F_0_*)) were calculated from the OJIP fluorescence transients according to^1^. Photosynthetic capacity, photosynthetic efficiency, light compensation point and dark respiration were calculated from the light dependency curves of oxygen evolution according to^2^.

| **Parameters** | **Strains** | | |  |
| --- | --- | --- | --- | --- |
|  | **IL** | **A250R** | **S264K** | |
| pg Chl*a*^4^ cell^-1^ | 0.0086±0.0019 | 0.005±0.0010 | 0.0035±0.0004 | |
| pg Chl*b*^4^ cell^-1^ | 0.0035±0.0008 | 0.0021±0.0004 | 0.0013±0.0002 | |
| pg Chl*(a+b)*^4^ cell^-1^ | 0.0103 ± 0.0011 | 0.0063 ± 0.0006 | 0.0043 ± 0.0003 | |
| Chl*a/b* | 2.49±0.04 | 2.54±0.07 | 2.50±0.03 | |
| Fv/Fm | 0.792 ± 0.005 | 0.753 ± 0.006 | 0.706 ± 0.006 | |
| 1-V_J_ | 0.56 ± 0.03 | 0.41 ± 0.02 | 0.23 ± 0.01 | |
| Photosynthetic capacity  (µmol O_2_ mg Chl^-1^ h^-1^) | 79 ± 2 | 55 ± 6 | 34 ± 2 | |
| Photosynthetic efficiency  (µmol O_2_ mg Chl^-1^ h^-1^)/ (µmol photon m^-2^ s^-1^) | 0.5 ± 0.1 | 0.4 ± 0.0 | 0.3 ± 0.0 | |
| Light compensation point  (µmol photon m^-2^ s^-1^) | 48 ± 5 | 59 ± 3 | 76 ± 4 | |
| Dark respiration  (µmol O_2_ mg Chl^-1^ h^-1^) | 21 ± 1 | 20 ± 1 | 27 ± 2 | |

^1^Strasser, R.J., Srivastava, A. & Tsimilli-Michael, M. The fluorescence transient as a tool to

characterize and screen photosynthetic samples. In Probing Photosynthesis: Mechanism,

Regulation and Adaptation (eds. Yunus, M., Pathre, U., Mohanty, P.) 443-480 (Taylor and

Francis, London, 2000).

^2^Walker, D. The use of the oxygen electrode and fluorescence probes in simple

measurements of photosynthesis,

http://citeseerx.ist.psu.edu/viewdoc/download?doi=10.1.1.129.2517&rep=rep1&type=pdf (1990).

^3^Lichtenthaler, H.K. Chlorophylls and carotenoids: pigments of photosynthetic biomembranes.

Methods Enzymol. 148, 350-382 (1987).

**Figure S2.** Carotenoid pigments accumulation in *C. reinhardtii* IL, A250R and S264K strains under physiological conditions. The content of zeaxanthin, anteraxanthin and violaxanthin (A), lutein (B) and β-carotene (C) per cell are presented. At least three separate runs in HPLC system were done per each strain. The values represent the average ± SD, n=9. P≤0.05 (Mann-Whitney U Test).

**
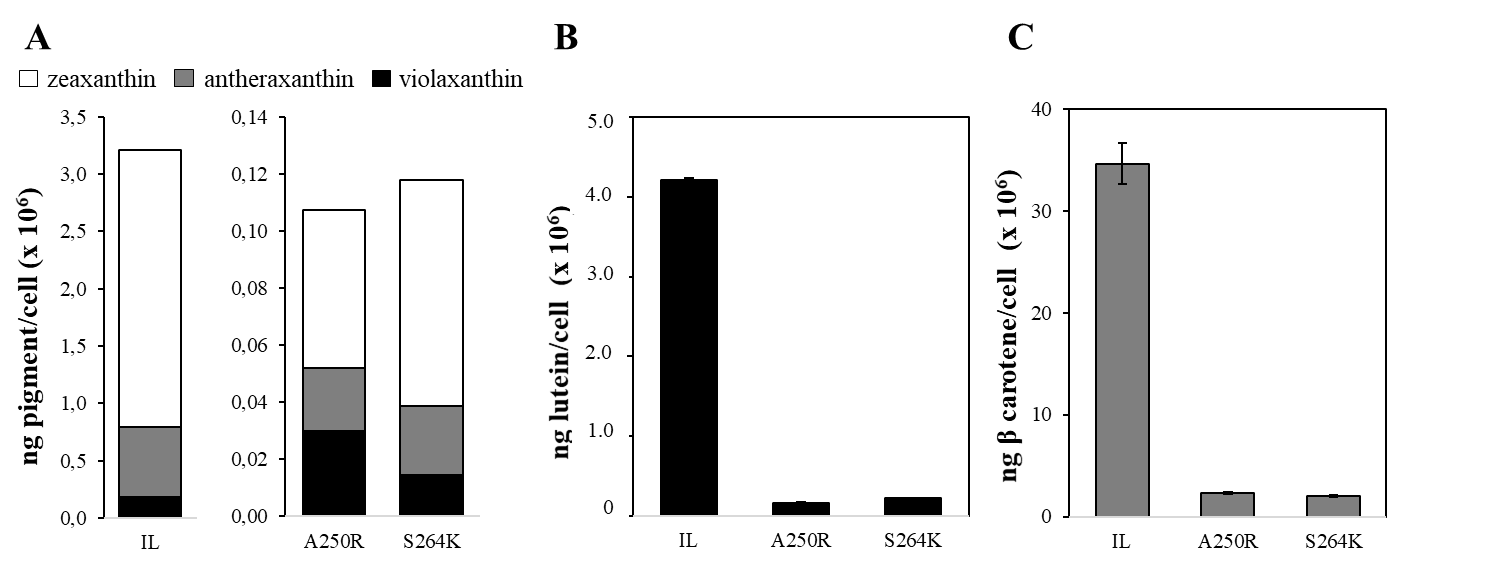
**

**Table S2.** NMR signal assignment and quantification of metabolite levels.

| **Num** | **Ppm** | **Assignment** | **Mean IL** | **SD_IL** | **Mean A250R** | **SD_A** | **Mean S264K** | **SD_S** |
| --- | --- | --- | --- | --- | --- | --- | --- | --- |
| 1 | 9.365 | NAD | 1.97 | 0.33 | 1.12 | 0.45 | 0.80 | 0.23 |
| 7 | 8.605 | AMP | 2.41 | 0.85 | 2.24 | 0.46 | 3.05 | 0.14 |
| 10 | 8.488 | Formic A | 28.60 | 10.37 | 28.86 | 7.61 | 38.95 | 5.45 |
| 31 | 7.574 | Uracil | 0.40 | 0.14 | 0.45 | 0.17 | 0.29 | 0.16 |
| 33 | 7.454 | Phe | 0.36 | 0.26 | 0.60 | 0.10 | 0.69 | 0.19 |
| 37 | 6.93 | Tyr | 0.32 | 0.19 | 0.65 | 0.21 | 0.69 | 0.14 |
| 38 | 6.536 | Fumaric A | 0.62 | 0.14 | 0.71 | 0.44 | 1.13 | 0.34 |
| 49 | 5.46 | Maltodextr | 2.85 | 1.25 | 1.26 | 0.26 | 3.20 | 1.00 |
| 88 | 3.037 | Lys | 1.56 | 0.96 | 1.94 | 0.48 | 2.45 | 0.76 |
| 102 | 2.708 | DHU | 0.50 | 0.23 | 0.50 | 0.18 | 1.02 | 0.56 |
| 104 | 2.687 | Malic A | 0.46 | 0.08 | 1.03 | 0.51 | 1.13 | 0.27 |
| 109 | 2.422 | Succinic A | 24.14 | 5.88 | 28.27 | 9.10 | 36.35 | 3.40 |
| 110 | 2.378 | Glu | 15.09 | 6.44 | 15.14 | 6.34 | 14.95 | 8.38 |
| 117 | 1.95 | Acetic A | 49.71 | 42.09 | 143.01 | 137.59 | 71.97 | 43.95 |
| 118 | 1.829 | Putrescine | 19.86 | 7.93 | 30.86 | 14.96 | 29.24 | 2.93 |
| 121 | 1.51 | Ala | 28.32 | 2.37 | 36.13 | 3.32 | 39.99 | 0.74 |
| 132 | 1.064 | Val | 3.65 | 0.40 | 3.46 | 0.10 | 3.24 | 0.24 |
| 133 | 1.029 | Ile | 0.57 | 0.08 | 0.61 | 0.17 | 0.61 | 0.05 |
| 134 | 0.984 | Leu | 5.26 | 1.24 | 4.87 | 0.51 | 5.49 | 0.62 |
|  | 1.35 | Thr | 3.89 | 0.74 | 2.68 | 0.89 | 2.29 | 0.25 |
|  | 1.34 | Lactic A | 35.45 | 16.93 | 43.39 | 16.25 | 25.84 | 10.10 |
| 28 | 7.80 |  | 0.18 | 0.06 | 0.41 | 0.13 | 0.24 | 0.10 |
| 29 | 7.78 |  | 0.35 | 0.05 | 1.04 | 0.43 | 0.66 | 0.19 |
| 30 | 7.73 |  | 0.47 | 0.21 | 1.97 | 1.70 | 1.11 | 0.40 |
| 41 | 6.10 |  | 0.57 | 0.21 | 0.21 | 0.06 | 0.22 | 0.07 |
| 52 | 4.62 |  | 7.41 | 2.38 | 3.60 | 0.61 | 5.77 | 2.84 |
| 63 | 3.78 |  | 94.67 | 7.21 | 85.12 | 18.53 | 81.61 | 5.20 |
| 91 | 3.00 |  | 0.39 | 0.06 | 0.67 | 0.12 | 0.60 | 0.25 |
| 94 | 2.96 |  | 0.53 | 0.03 | 0.87 | 0.07 | 0.65 | 0.07 |
| 99 | 2.82 |  | 2.13 | 0.17 | 0.78 | 0.87 | 0.66 | 0.19 |
| 112 | 2.19 |  | 16.19 | 1.66 | 12.05 | 1.33 | 11.48 | 1.15 |
| 125 | 1.25 |  | 0.71 | 0.26 | 1.19 | 0.11 | 0.83 | 0.18 |
| 126 | 1.23 |  | 1.00 | 0.27 | 1.63 | 0.36 | 1.12 | 0.02 |
| 127 | 1.21 |  | 0.30 | 0.08 | 0.59 | 0.12 | 0.62 | 0.27 |

**Figure S3.** An expanded PCA scores plot region where A250R and S264K sample scores are located.

**Figure S4.** Correlation plots for β-carotene/α-carotene (A) and chlorophylls a and b (B).

**Table S3.** Ratios (A250R/S264K) of mean values of pigments. The values are reported only for statistically significant (p<0.05) differences. When the level of a specific pigment is higher in S264K than in A250R, the corresponding value is highlighted by gray background color.

| **Type** | **Time** | **VIO** | **ANTER** | **CHL b** | **LUTEIN** | **ZEA** | **CHL a** | **α-CAR** | **β-CAR** |
| --- | --- | --- | --- | --- | --- | --- | --- | --- | --- |
|  | T0 | 0.70 |  | 0.79 | 0.74 | 2.05 | 0.75 |  | 1.12 |
| Control | T15 | 3.42 |  | 2.54 | 2.63 | 7.82 | 2.11 | 2.98 | 1.94 |
| Control | T30 | 1.75 | 2.67 | 1.75 | 6.29 | 7.51 | 1.59 | 3.87 | 4.68 |
| Control | T90 | 1.61 | 1.40 | 1.41 | 1.56 | 1.53 | 1.37 | 1.57 | 1.84 |
| HL/HT | T15 |  | 1.24 | 2.14 | 1.55 |  | 1.91 |  | 1.30 |
| HL/HT | T30 | 0.34 | 1.85 | 0.20 | 0.71 |  | 0.45 | 3.60 | 2.29 |
| HL/HT | T90 | 1.42 | 5.50 | 0.74 | 1.86 | 4.48 | 1.17 | 4.30 | 2.12 |

**Table S4.** Sequences of primers used in the two-step PCR for the site-directed mutagenesis. The modified nucleotides in the mutagenic primers (e.g. corresponding to Ala→Arg amino acid substitution in position 250 in D1 protein) are pointed out.

| **1st PCR** | | **2nd PCR** | |
| --- | --- | --- | --- |
| **Primers** | **Sequence (5’ → 3’)** | **Primers** | **Sequence (5’ → 3’)** |
| **outer for** | GGTGCTGTAATCCCAACTTCT |  |  |
| **outer rev** | CTAGAGTTAGTTGAAGCTAAGTCTAGAGGGA |  |  |
| **A250 rev** | TACAATGTTGTAAGTTTCTTCTTC | **A250R mut-rev** | CGACCAAAGTAACCATGAGC**ACG**TACAATGTTGTAAG |
| **A250 for** | GCTCATGGTTACTTTGGTC | **A250R mut-for** | CAACATTGTA**CGT**GCTCATGGTTACTTTGGTCGTC |
| **S264 rev** | AGCGTATTGGAAGATTAG | **S264K mut-rev** | GAACGAGAGTTGTTGAA**TTT**AGCGTATTGGTTC |
| **S264 for** | TTCAACAACTCTCGTTC | **S264K mut-for** | CGTCTAATCTTCCAATACGCT**AAA**TTCAACAACTC |

**Table S5.** Specific primers used for qRT-PCR analysis and amplified fragments size

| **Gene** | **Primer sequence (5'→3')** | **Amplified fragment size (cDNA bp)** | ***C. reinhardtii***  ***loci*** |
| --- | --- | --- | --- |
| *psy* | TGGATGAGCTGGACAAGT  GTCCGTGAAGTATTGCCG | 140 | AY604702 |
| *pds* | ACCATGACTGAGCTGGAG CTTGTACTTGCGGATCTTGG | 78 | XM_001690807 |
| *lyc-β* | TGACGCTGTTCTGGAAGA CTCCTTGAGCGACATTGT | 140 | AY860818 |
| *lyc-ε* | CAAGTTCCTGCGCTATGA GCCTGAAATCCATGAAGGTC | 128 | XM_001696477 |
| *chy-β* | CAACCTGCCGTACATGAA CCCAGAAACATGCCGAA | 90 | XM_001698646 |
| *zep* | AGGACAGCCGCATTTTG TCAACCCACTTGCTCCA | 112 | XM_001701649 |
| *vdr1* | CCTTCTACTTGTCGGTATTGG TTCTCATCGCAGTCCACA | 73 | XM_001694990 |
| *mpbq-mt* | CACCCCTACTTCATCTCCAT GTGTTCTTGTTCCAGTCCTC | 95 | XM_001692671 |
| *hst1* | ACCAGCCTCTACACCTTT TGTACACGCCGAAGTTGA | 136 | XM_001695289 |
| *rack1* | TGCAAGTACACCATTGGC CCAGACCTTGACCATCTTGT | 123 | XM_001698013 |

**Figure S5. A**. Diameter of IL, A250R and S264K cells (µm) with relative standard deviations, and the Feret diameters, defined as the longest distance between two points along the perimeter of a particle. **B**. Chl*a* content per cell in *C. reinhardtii* strains. The average values of three independent experiments are shown. The ratio Chl*a*/cell was calculated, using the average value for the chlorophyll content and the cell number in each independent experiment. For each independent experiment three internal repetitions were done. The values represent the average ± SD, n=9. P≤0.05 (Mann-Whitney U Test).


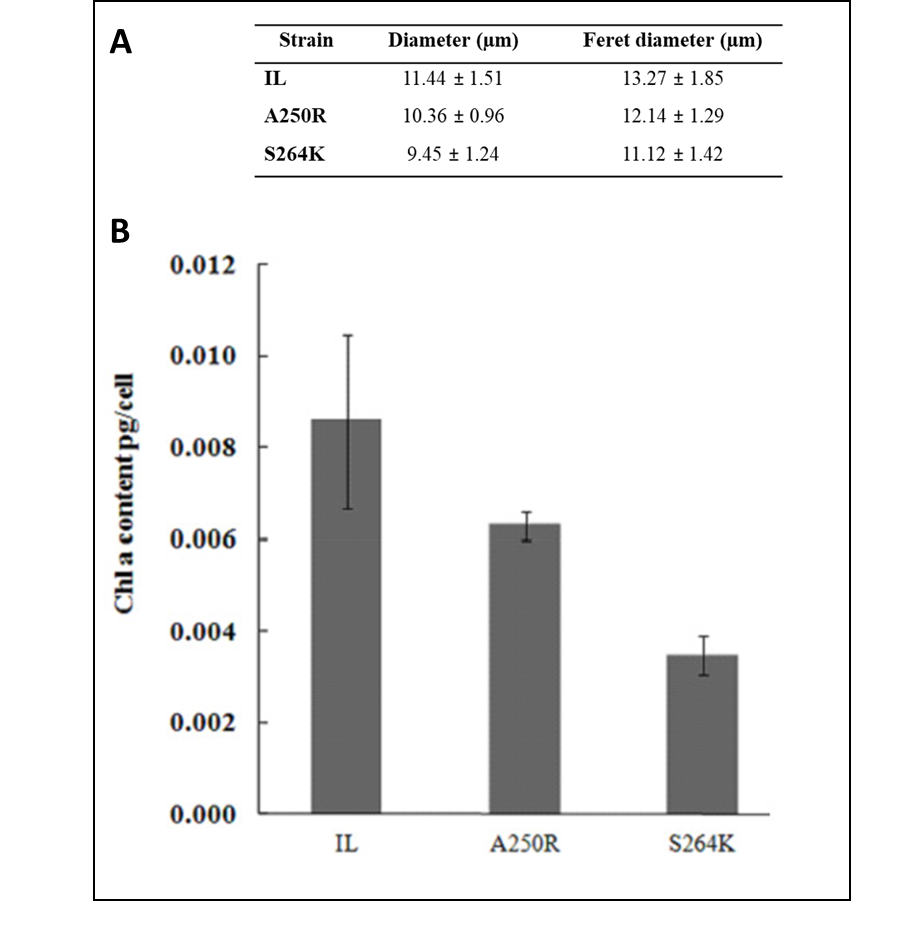

Supplement: Supplementary file 1 — Supplementary File [file 41598_2018_33146_MOESM1_ESM.docx]
